# A ROS‐Responsive Lipid Nanoparticles Release Multifunctional Hydrogel Based on Microenvironment Regulation Promotes Infected Diabetic Wound Healing

**DOI:** 10.1002/advs.202403219

**Published:** 2024-09-23

**Authors:** Hao Yang, Dongming Lv, Shanqiang Qu, Hailin Xu, Shuting Li, Zhiyong Wang, Xiaoling Cao, Yanchao Rong, Xiaohui Li, Honglin Wu, Yongfei Chen, Jiayuan Zhu, Bing Tang, Zhicheng Hu

**Affiliations:** ^1^ Department of Burn and Wound Repair First Affiliated Hospital of Sun Yat‐sen University Guangzhou 510080 China; ^2^ Department of Neurosurgery Nanfang Hospital of Southern Medical University Guangzhou 510515 China; ^3^ Department of Dermatology Dermatology Hospital of Southern Medical University Guangzhou 510091 China; ^4^ Department of Plastic Surgery First Affiliated Hospital of Sun Yat‐sen University Guangzhou 510080 China; ^5^ Department of Joint Surgery The Third Affiliated Hospital of Guangzhou Medical University Guangzhou 510150 China

**Keywords:** antibacterial, anti‐inflammation, antioxidation, diabetic wound, multifunctional hydrogel

## Abstract

The continuous imbalance of the diabetic wound microenvironment is an important cause of chronic nonhealing, which manifests as a vicious cycle between excessive accumulation of reactive oxygen species (ROS) and abnormal healing. Regulating the microenvironment by suppressing wound inflammation, oxidative stress, and bacterial infection is a key challenge in treating diabetic wounds. In this study, ROS‐responsive hydrogels are developed composed of silk fibroin methacrylated (SFMA), modified collagen type III (rCol_3_MA), and lipid nanoparticles (LNPs). The newly designed hydrogel system demonstrated stable physicochemical properties and excellent biocompatibility. Moreover, the release of antimicrobial peptide (AMP) and puerarin (PUE) demonstrated remarkable efficacy in eradicating bacteria, regulating inflammatory responses, and modulating vascular functions. This multifunctional hydrogel is a simple and efficient approach for the treatment of chronic diabetic infected wounds and holds tremendous potential for future clinical applications.

## Graphical abstract

The prevalence of diabetes‐related chronic non‐healing wounds presents a daunting challenge to healthcare professionals worldwide. In the study, a hydrogel system is designed that responds dynamically to the unique microenvironment of diabetic wounds. By integrating silk fibroin methacrylated (SFMA), modified collagen type III (rCol_3_MA), lipid nanoparticles (LNPs), antimicrobial peptide (AMP), and puerarin (PUE), the hydrogel embodies the convergence of cutting‐edge biomaterials science and compassionate patient care.

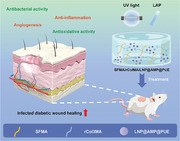

## 1 Introduction

The incidence of diabetes is increasing annually, with type II diabetes affecting younger individuals.^[^ ^1^ ^]^ Chronic nonhealing wounds, a common complication of diabetes, pose significant risks, such as prolonged hospitalization, economic burden, infection, gangrene, amputation, and even mortality.^[^ ^2^ ^]^ However, current interventions have not yielded substantial improvements in these outcomes.^[^ ^2^ ^]^ Therefore, it is imperative to explore more efficacious treatment modalities for enhancing chronic wound healing in individuals with diabetes.

Chronic nonhealing wounds in diabetic conditions are highly susceptible to bacterial infections, leading to the formation of exudates and biofilms that impede the wound‐healing process.^[^ ^3^ ^]^ The main feature of chronic wounds is the stagnation of the inflammatory period, which manifests as an imbalance of inflammatory factors, the inhibition of cell proliferation, and angiogenesis disorders. These manifestations damage the wound microenvironment and lead to the accumulation of reactive oxygen species (ROS), and excessive accumulation of ROS aggravates the abnormal healing pathway.^[^ ^4^ ^]^ Therefore, improving the wound microenvironment by inhibiting the inflammatory response, inducing angiogenesis, and eliminating bacterial infection are crucial for the treatment of chronic diabetic wounds.^[^ ^5^ ^]^ However, the current primary clinical treatment strategy still relies on a single‐target approach, which may be both expensive and ineffective. Therefore, there is a need for a multifunctional and efficient treatment method to address practical clinical issues.^[^ ^6^, ^7^ ^]^ In recent years, multifunctional hydrogels have garnered significant attention because of their anti‐inflammatory, antibacterial, hemostatic, response stimulation, and drug delivery capabilities.^[^ ^8^ ^]^ Owing to their specific porous structure and swelling properties, hydrogels have unique advantages in wound healing applications. Their excellent permeability is conducive to the absorption of wound exudate, maintaining a moist environment, controlling drug release, and ultimately promoting wound healing.^[^ ^9^ ^]^ Therefore, the focus of research in the treatment of chronic diabetic wounds is developing a multifunctional hydrogel loaded with effective drugs.

The inflammatory phase represents a critical period for diabetic‐infected wounds, with early suppression treatment being crucial for determining wound closure rates and scar formation. Therefore, the design of multifunctional hydrogels should prioritize the regulation of early inflammatory environments. In recent years, antimicrobial peptides (AMPs) have emerged as promising therapeutic agents for combating bacterial infections and promoting skin regeneration because of their antibacterial, angiogenic, and immunomodulatory properties.^[^ ^10^, ^11^, ^12^ ^]^ The immunomodulatory effects of traditional Chinese medicine have also been documented.^[^ ^13^, ^14^, ^15^ ^]^ Puerarin (PUE), which is extracted from the root of Pueraria lobata, is a traditional Chinese medicine that has anti‐inflammatory, antioxidative, and oxidative stress relief properties. It has been utilized in the treatment of myocardial infarction, hypertension, and diabetes.^[^ ^16^, ^17^, ^18^ ^]^ Additionally, PUE exhibits wound‐healing activity, and hydrogels formulated with PUE have demonstrated therapeutic effects on skin wounds.^[^ ^19^ ^]^ Biomaterial‐based therapeutic strategies offer a promising approach to regulate the spatiotemporal delivery of active drugs, thereby mitigating side effects, enhancing potency, and reducing the frequency and quantity of doses needed. These properties ultimately improve the therapeutic efficacy in tissue regeneration. Considering the persistently elevated ROS levels in the diabetic wound microenvironment,^[^ ^20^, ^21^, ^22^ ^]^ the design of a ROS‐responsive vehicle for drug transport and release could further improve drug utilization and therapeutic efficacy.

It is of paramount importance to establish a drug delivery system that accommodates diverse characteristics and enables sustained release, thereby enhancing the wound microenvironment and facilitating diabetic wound healing. Liposomes are nanocarriers composed of bilayered structures and are characterized by facile preparation, versatility, high biocompatibility, and biodegradability.^[^ ^23^, ^24^ ^]^ Owing to the unique structure of liposomes, they can encapsulate both hydrophilic and hydrophobic molecules.^[^ ^25^ ^]^ Phenylboronic Acid (PBA) is a class of hydrophobic small‐molecule compounds that are highly sensitive to ROS because of the formation of boronic ester bonds. At low concentrations of hydrogen peroxide, ROS can act as nucleophiles to selectively coordinate with PBA. In acidic environments with high levels of ROS, carriers based on boronic ester bonds may degrade, resulting in the intelligent release of drugs.^[^ ^26^ ^]^ Therefore, PBAs can be used to construct an ROS response delivery system. Dextran (DEX) is a hydrophilic natural polysaccharide that has extensive applications in the fields of cargo transportation, medical implants, wound dressings, and tissue engineering because of its excellent biocompatibility and biodegradability.^[^ ^27^ ^]^ By coupling 4‐(hydroxymethyl)‐phenylborate pinalol ester (PBAP) to the hydrophilic DEX side chain via a carbonyl diimidazole (CDI) reaction, ROS‐responsive liposomes can be prepared through hydrophilic and hydrophobic self‐assembly. Liposomes can rapidly disintegrate and dissolve in water upon exposure to ROS, thereby facilitating drug release.

The selection of appropriate hydrogel materials is also important. Type III collagen is an extracellular matrix protein that confers elasticity and structural support to the skin.^[^ ^28^ ^]^ Owing to its high biological activity, type III collagen is becoming increasingly popular in biomedical fields such as cosmetics, wound healing, and artificial blood vessel research.^[^ ^29^ ^]^ The production of type I collagen can be regulated by type III collagen, which accelerates the fibrogenesis process and regulates its structural and functional properties.^[^ ^30^ ^]^ The application of biotechnology enables the creation of analog proteins and peptides that can effectively mitigate immune responses.^[^ ^31^ ^]^ The production of recombinant type III collagen protein (rCol_3_), however, relies on genetically modified microorganisms that yield products with a molecular structure and nature that more closely resemble human collagen, making them suitable for large‐scale manufacturing.^[^ ^32^ ^]^ To obtain a photopolymerizable material, methacrylic anhydride (MA) can be utilized for the chemical modification of collagen, resulting in the formation of methylacrylated collagen (ColMA).^[^ ^33^ ^]^ Moreover, silk fibroin (SF), a widely utilized material in the realm of biomedicine, has favorable attributes, such as excellent biocompatibility, degradability and high tensile strength.^[^ ^34^ ^]^ Silk fibroin glyceryl methacrylate (SFMA), which is formed by modifying SF with glyceryl methacrylate (GMA), enhances hydrogel stability and the degree of optical crosslinking.^[^ ^35^ ^]^ An ideal hydrogel platform can be constructed based on these respective advantageous features.^[^ ^36^ ^]^

**Figure 1:**
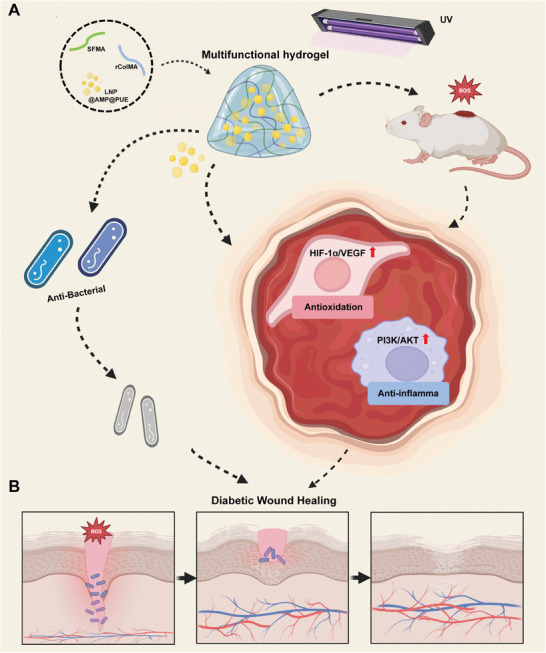
Diagrams displaying A) the formation of the SFMA/rColMA/LNP@AMP@PUE hydrogel and B) the mechanisms of accelerated wound healing.

In this study, we developed a novel multifunctional hydrogel to accelerate diabetic wound healing by combining lipid nanoparticles (LNPs) loaded with two different drugs (AMP and PUE) to achieve sustained drug release in the ROS microenvironment to exert antibacterial, anti‐inflammatory, and proangiogenic effects (**Figure** **1**). We characterized the physical and chemical properties of the multifunctional hydrogel and evaluated its ability to repair it in vitro and in vivo. All the data presented in this study indicate that the SFMA/rColMA/LNP/@AMP@PUE hydrogel is a promising dressing for promoting wound repair in chronic diabetic wounds.

## 2 Experimental Section

### 2.1 Materials

The materials used in this work are listed in Table S1 (Supporting Information).

### 2.2 Preparation of Modified Collagen Type III (rCol_3_MA) and Silk Fibroin Methacrylated (SFMA)

One gram of rCol_3_ was weighed and dissolved in 50 mL of distilled water. After full dissolution, the pH was adjusted to 7, 1 mL of methacrylic anhydride was added, and the mixture was allowed to react at room temperature for 24 h. The modified collagen type III (rCol_3_MA) was obtained by dialysis with distilled water for 3‒5 days (molecular weight interception: 3500), centrifugation at 10000 rpm for 5 min, filtration on neutral filter paper, and freeze‐drying at −80 °C.

Degummed SF was obtained by immersing 10 g of silkworm cocoon in 1 L of 0.05 M Na_2_CO_3_ solution, boiling it at 100 °C for 30 min, and washing it several times with distilled water. The degummed SF was subsequently dried in a drying oven for 36 h. To obtain SFMA, 10 g of degummed SF was dissolved in a 9.3 m lithium bromide (LiBr) solution at 60 °C for 1 h and stirred with a magnetic stirrer, after which 6 mL of GMA was slowly added in a dropwise manner. After 8 h of reaction, the solution was filtered through a filter cloth, and the salt was removed and dialyzed with distilled water for 7 days in a dialysis bag. The resulting SFMA solution was freeze‐dried for 36 h to obtain spongy SFMA.

### 2.3 Preparation of ROS‐Responsive Liposomes Loaded with AMP and PUE

#### 2.3.1 Synthesis of PHB‐DEX

PBAP (0.339 g, 31.5 mm) was dissolved in 46 mL of CH_2_Cl_2_. CDI (0.469 g, 62.9 mm) was added, and the mixture was stirred for 30 min. The mixture was diluted in ethyl acetate (200 mL) and washed with deionized water (3 × 10 mL). The organic matter was washed with saline (1 × 10 mL), dried over MgSO_4_, and concentrated under a vacuum to obtain a pure white solid, PBAP‐CDI.

DEX (molecular weight = 10500 g mol^−1^, 90 mg, 0.0086 mmol) was dissolved in anhydrous DMSO (1.575 mL), after which 4‐dimethylamino‐pyridine (DMAP) (149.3 mg, 1.22 mmol) was added, followed by CDI‐activated PBAP (364.6 mg, 1.11 mmol, 2.0 mol/L). The mixture was stirred overnight, and the modified DEX was subsequently precipitated in deionized water (35 mL). The products were separated by centrifugation for 15 min, and the resulting pellet was washed with deionized water (2 × 35 mL). Phenylborate‐modified dextran (PHB‐DEX) nanoparticles were collected via lyophilization.^[^ ^37^ ^]^

#### 2.3.2 Preparation of ROS‐Responsive Lipid Nanoparticles (LNPs) Loaded with AMP and PUE

Twelve milligrams of PHB‐DEX dissolved in 200 µL of DMSO was weighed, and then 7.4 mg of hydrogenated soy phosphatidylcholine (HSPC), 16.5 mg of cholesterol (CHO‐HP), and 12 mg of PUE dissolved in 1.8 mL of ethanol were weighed, dispersed via ultrasonication, and fully dissolved. The ROS‐responsive LNP@AMP@PUE liposome solution was obtained by injecting 10 mL of violently stirred AMP aqueous solution (1.2 mg mL^−1^) and stirring for 15 min, followed by sonication for 15 min (210 W).

### 2.4 Preparation of Hydrogels

The SFMA/rColMA hydrogel was prepared by mixing 20% SFMA and 4% rCol_3_MA solutions at a volume ratio of 1:1 followed by cross‐linking (30 s) under conditions of LAP (1 mg ml^−1^) and UV light (20 W, 405 nm). The SFMA/rColMA/LNP@AMP@PUE hydrogel was obtained by mixing 100 µL of LNP@AMP@PUE liposome solution into 1 mL of 20% SFMA and 4% rCol_3_MA solution followed by cross‐linking under LAP and UV light.

### 2.5 Physical and Chemical Property Testing of the Hydrogels

#### 2.5.1 FTIR

Eight milligrams of sample (SF, SFMA, rCol_3_, or rCol_3_MA) and an appropriate amount of dry potassium bromide powder (mass ratio of ≈5%) were weighed in an agate mortar and ground thoroughly to ensure even mixing. Next, an appropriate amount of ground sample powder collected and pressed (vacuum pressure of 20 mmHg, pressed for 5 min) to obtain a sample sheet. The scanning range was set to 4000–500 cm^−1^, and a Fourier transform infrared spectrometer was used for detection.

#### 2.5.2 H‐NMR

Eight milligram samples (SF, SFMA, rCol_3_, and rCol_3_MA) were weighed, dissolved in deuterium‐heavy water, and ultrasonically dispersed. After the solution was completely dissolved and clarified, it was loaded into a clean nuclear magnetic tube. The nuclear magnetic structure was determined at room temperature using a nuclear magnetic resonance spectrometer, and map analysis was performed using MestReNova software.

#### 2.5.3 Scanning Electron Microscopy (SEM)

The hydrogel scaffolds (SFMA/rCol_3_MA and SFMA/rCol_3_MA/LNP@AMP@PUE) were frozen at −80 °C for prefreezing. After freeze‐drying, gold was sprayed onto the surface of the hydrogel for 30 s, and the surface morphology of the hydrogel was observed via SEM.

#### 2.5.4 Swelling Ratio

The dry weight (W_dry_) of different samples of the hydrogel scaffolds was measured, after which the samples were immersed in 37 °C PBS buffer (pH 7.4). The mixture was removed at different time points (1, 2, 3, 4, 5, 8, and 10 h), the surface water was dried with filter paper, and then the hydrogel was weighed to obtain its weight (W_swollen_). The water absorption ratio (Q) of the hydrogel can be calculated as follows: Q = (W_swollen –_ W_dry_)/W_dry_ × 100%.

#### 2.5.5 Rheological Properties

After the hydrogels of different samples (300 µL) were released, the rheology was measured by turning a stainless steel plate with a diameter of 25 mm. G' represents the elastic modulus, and G’’ represents the viscous modulus of the sample. Dynamic strain scanning was performed at room temperature from 0.1 rad/s to 10 rad/s to determine the linear viscoelastic range of the hydrogel, and the change curves of the storage modulus (G') and loss modulus (G’’) were recorded.

#### 2.5.6 Compression Strength

The cylindrical hydrogel sample was placed in a universal testing machine and compressed at a constant rate. The stress value when the strain reached 80% was recorded, and the compression strength was calculated according to the following formula: P = F/S, where F is the recorded stress value, and S is the cross‐sectional area of the cylindrical sample.

#### 2.5.7 Degradation Performance (Degradation Rate)

The 10%SFMA/2%rCol_3_MA/LNP@AMP@PUE hydrogel was freeze‐dried and weighed as W_0_; then, the initial sample was soaked in a PBS solution containing 1000 U ml^−1^ lysozyme in a constant‐temperature shaker (37 °C, 70 rpm). At the measured time points, the hydrogel was removed, washed with ultrapure water, and freeze‐dried. The mass was accurately weighed after drying, and the weight (W_t_) was recorded. The mass residue rate of the stent was calculated using the following formula: mass residue rate (%) = W_t_/W_0_ × 100%.

### 2.6 Drug Release In Vitro

One milliliter of the hydrogel was placed in 5 ml of PBS‐H_2_O_2_ (0.1 mm) and PBS, followed by incubation at 37 °C, collection of the supernatant at the same time points (1, 3, 6, 12, 24, 48, 72 and 96 h), and filling with the same volume of solution. The AMP (205 nm) and PUE (306 nm) contents of the collected supernatants were measured using a UV spectrophotometer to calculate the cumulative release rate. The cumulative release formula was as follows: cumulative release rate (%) = amount of drug released/total drug content × 100%.

### 2.7 Characterization of Antibacterial Properties In Vitro

*Escherichia coli* and *Staphylococcus aureus* were resuscitated and cultured to the logarithmic growth phase and centrifuged, and the bacteria were collected and resuspended in normal saline to a final bacterial concentration of 1×10^8^ CFU mL^−1^. After the hydrogel was prepared with sterile normal saline, the volume of hydrogel in each well was 200, and 200 µL of normal saline was added to the control group. Subsequently, 100 µL of diluted bacterial suspension and 1 mL of Luria–Bertani (LB) liquid medium were added to each well, incubated at 37 °C for 4 h, gradient diluted, coated on LB agar plates, and counted. After being fixed with 2.5% glutaraldehyde for 4 h, the LB medium was removed via centrifugation. After washing with PBS and pure water, the bacterial suspension dispersed in pure water was dropped onto a single‐crystal silicon wafer. After natural air drying, the bacterial suspension was sprayed with gold for 30 s, and the bacterial morphology was captured via a scanning electron microscope.

### 2.8 Network Pharmacological Analysis

#### 2.8.1 Dataset Selection and Processing

The expression profile of GSE80178 was obtained from the Gene Expression Omnibus (GEO) database, which consists of 6 samples of diabetic foot ulcers (DFUs) and 3 samples of normal skin tissues. Differentially expressed genes (DEGs) were identified on the basis of a fold change in expression >2.0 and an FDR < 0.05. The xCell algorithm was applied to calculate the scores for the microenvironment and evaluate the cell composition.

#### 2.8.2 PUE‐Related Target Genes

We used the Traditional Chinese Medicine Systems Pharmacology Database and Analysis Platform (TCMSP), Comparative Toxicogenomics Database (CTD) and SwissTargetPrediction database (STP) to search for potential therapeutic targets of PUE. For gene set functional enrichment analysis, we used the KEGG rest API to obtain gene annotations of the latest KEGG pathway and used the R software package clusterProfiler (version 3.14.3) to obtain the results of the gene set enrichment.

### 2.9 In Vitro Cellular Experimentation Section

#### 2.9.1 Cell Culture

The human monocyte cell line THP‐1, the human umbilical vein endothelial cell line HUVEC, and the mouse fibroblast cell line L929 were purchased from the Typical Culture and Preservation Committee of the Cell Bank, Chinese Academy of Sciences. All the above cells were cultured in complete medium maintained at 37 °C with 5% carbon dioxide and subcultured or exchanged as appropriate. THP‐1 cells can differentiate into macrophages after treatment with phorbol 12‐myristate 13‐acetate (PMA). The cells that were cocultured with different hydrogels in this study were generally at passages 3−5.

#### 2.9.2 CCK‐8 Test

Cell proliferation was determined via a cell counting kit‐8 (CCK8) assay (APExBIO, USA). Cells in the logarithmic growth phase were seeded into 96‐well plates, and CCK8 solution was added to the medium, followed by incubation at 37 °C for 2 h. The absorbance was measured three times at 450 nm using an enzyme immunoassay analyzer.

#### 2.9.3 Cell Migration Assay

The migration ability of the fibroblasts was evaluated via a Transwell assay. The cell suspensions were diluted in serum‐free medium and seeded into the upper chamber of a 24‐well plate (Corning, USA) containing an 8.0‐µm polycarbonate membrane. Complete medium was added to the lower chamber. After 24 h, the upper membrane cells were removed, and the migrated cells on the lower surface were stained with crystal violet (Beyotime, China). After washing with PBS, the stained cells were counted via light microscopy.

#### 2.9.4 Mitochondrial Membrane Potential (φM)

The lipophilic cationic dye JC‐1 (MCE, USA), which is selectively taken up by mitochondria, was employed to visualize the mitochondrial membrane potential. By coincubating HUVECs with JC‐1 at a final concentration of 2 µm for 15 min, changes in the mitochondrial membrane potential could be evaluated through the observation of alterations in the fluorescence intensity of mono‐ and multimers via fluorescence microscopy.

#### 2.9.5 Wound‐Healing Assay

HUVECs were cultured in 6‐well plates containing complete medium, and the cell surface was scraped vertically with a 200 µl plastic pipette tip to create a wound. The cell debris was removed by washing three times with PBS, and then medium containing 1% FBS was added. At time points (0 h and 24 h), a specific area within the scratch was captured via a microscope equipped with a digital camera.

#### 2.9.6 Tube Formation Assay

HUVECs were seeded onto 48‐well culture plates precoated with Matrigel (BD Biosciences, USA). After a 6‐h incubation at 37 °C, images were captured under magnification using an inverted microscope.

#### 2.9.7 Quantitative Real‐Time Polymerase Chain Reaction (qRT‒PCR)

The expression levels of VWF, Vcam1, CD31, CD206, ARG‐1, IL10, CD80, iNOS and IL1β were detected via qRT‒PCR. RNA was extracted with a TRIzol kit (Life Technologies, USA), and first‐strand cDNA was synthesized via reverse transcription using gDNAEr reagent kit (Takara, Japan) according to the manufacturer's protocol. qRT‒PCR was performed using a Bestar®SybrGreen qPCR Master mixture (DBI, Germany). The sequences of primers used for qRT‒PCR are shown in Table S2 (Supporting Information).

#### 2.9.8 Flow Cytometry

THP‐1 cells were harvested, washed with PBS, and resuspended in flow cytometry buffer (1× PBS buffer containing 1% FSA) supplemented with anti‐CD163 and anti‐CD86 antibodies (BD Biosciences, USA) at 20 °C for 30 min. The cells were subsequently washed again, resuspended, and analyzed via flow cytometry (BD Biosciences, USA) according to the manufacturer's instructions.

#### 2.9.9 Enzyme‐Linked Immunosorbent Assay (ELISA)

To detect the release of macrophage‐derived proinflammatory cytokines (TNFα, IL‐1β, and IL‐6) and anti‐inflammatory cytokines (TGFβ, bFGF, and IL‐10), the assay was performed using a human ELISA kit (Meimian, China), and the absorbance at 450 nm was measured with a microplate reader within 15 min after the reaction was stopped.

#### 2.9.10 Transcriptome Sequencing

After the treated cells were collected, TRIzol was used for cell lysis and total RNA extraction. The cDNA libraries were subjected to sequencing on the Illumina sequencing platform by Genedenovo Biotechnology Co. Ltd. (Guangzhou, China). DEGs were determined by FDR<0.05 and |log2FC|>1. Bioinformatic analysis was performed via OmicsMart, a real‐time interactive online platform for data analysis (http://www.omicsmart.com).

#### 2.9.11 Western Blot

The proteins from the cultured cells were extracted with RIPA lysis buffer (MIK, China). The total concentration of protein collected from the cells was calculated with a BCA protein assay kit (Solarbio, China). Protein samples were separated via SDS‒PAGE and transferred to PVDF membranes (Millipore, USA). After the membrane was blocked, it was incubated overnight at 4 °C with primary antibodies against [GAPDH; ab181602], [HIF‐1α; ab179483], [p‐AKT; ab81283], [AKT; ab8805], and [VEGF; ab214424]). An HRP‐labeled secondary antibody was used for the detection of the antibody‐reactive protein. The protein signal was detected via chemiluminescence western blotting detection solution (Bio‐Rad, USA).

### 2.10 Establishment of a Rat Wound Model and Evaluation of Treatment

#### 2.10.1 Establishment of a Comprehensive Rat Model for Full‐Thickness Skin Injury and Infection

Male SD rats (8–10 weeks old) were intravenously injected with streptozotocin (65 mg kg^−1^). Three to four days after injection, blood glucose was measured every 2 days until the blood glucose level exceeded 17 mm. Diabetic rats were anesthetized via an intraperitoneal injection of 1% sodium pentobarbital (45–60 mg kg^−1^). The hair on and around the back of each rat was stripped with an animal shaving device and depilated with depilation cream, and the exposed skin was sterilized with povidone‐iodine solution. The skin of the back was gently lifted 5 mm on both sides of the median line of the back, and round wounds with a diameter of 12 mm were cut off. A mixture of *E. coli and S. aureus* (40 µL, 1×10^8^ CFU mL^−1^) was added to each wound, and the experiment was carried out 24 h after infection. The infected rats were randomly divided into six groups. Different materials were used to seal the wounds, and 3m Tegaderm waterproof tape was used to cover the outer layer for fixation. Wound healing was observed at 3, 7, 12, and 18 days after the operation, and sterile gauze or hydrogel dressing was not replaced during this period.

#### 2.10.2 Evaluation of Antibacterial Activity In Vivo

The wound samples from Day 3 were collected, and 1 mL of saline was added and ground. The ground liquid was diluted in a saline gradient, coated in mannitol sodium chloride agar medium and gram‐negative bacteria selective medium, and incubated in inverted culture (37 °C, 5% CO_2_) for 24 h before counting.

#### 2.10.3 Histological Evaluation

Wound samples at Days 7, 12 and 18 were collected, fixed with 4% methanol in PBS, and then stored at 4 °C. The samples were then dehydrated with an alcohol concentration gradient and embedded in paraffin. Finally, hematoxylin‒eosin (HE) and Masson's trichrome staining were used to stain the samples. These tissue sections were subsequently carefully examined under a light microscope.

#### 2.10.4 Immunofluorescence Staining

The paraffin sections were deparaffinized and washed three times in xylene for 15 min, followed by two 5 min washes with absolute ethanol, one 5 min wash with 85% and 75% ethanol, and a final rinse with distilled water. The slides were then incubated with primary antibodies overnight at 4 °C. After three 5‐min rinses with PBS (pH 7.4), the slides were incubated with secondary antibodies for 50 min at room temperature. After rinsing as described above, the cells were subsequently incubated with DAPI for 10 min in the dark. The resulting sections were then viewed under a laser scanning confocal microscope. The antibodies used are shown in Table S3 (Supporting Information).

### 2.11 Ethics Approval and Consent to Participate

This study was approved by the ICE for Clinical Research and Animal Trials for the First Affiliated Hospital of Sun Yat‐sen University. Approval No: (2023)173.

### 2.12 Statistical Analysis

The statistical trends from different groups were analyzed by one‐way ANOVA with Tukey's posttest (GraphPad Prism 9.5). The data from at least three individual experiments are presented as the means ± standard deviations (means ± SDs). Data were considered statistically significant when *P* < 0.05.

## 3 Results and Discussion

### 3.1 Chemical Structure Analysis of LNPs and Hydrogels

To construct ROS‐responsive LNPs, we first synthesized amphiphilic polymers. As shown in Figure S1A (Supporting Information), in the infrared spectrum of PHB‐DEX, the (carbonate) ester group ─O─C═O─O─ (1748 cm^−1^) was introduced after the reaction of DEX with PBAP‐CDI occurred. The characteristic absorption peaks at 1560, 1500 and 860 cm^−1^ were attributed to para‐substituted benzene rings. Figure S1B (Supporting Information) shows that hydrogen on the benzene ring appears at signals of 7.33 and 7.64 ppm in the PHB‐DEX spectrum, which indicates an effective reaction between PHB and DEX and that PHB‐DEX was successfully synthesized. PHB‐DEX could be hydrolyzed by breaking ether bonds under H_2_O_2_‐induced oxidative stress, which then triggered drug release. In conclusion, we successfully prepared biparental molecules that could form structurally stable liposomes under nonoxidative stress conditions, and the purpose of this study was to use LNPs as carrier‐loaded drugs for controlled release of ROS.

Moreover, to obtain a good wound dressing, we synthesized a hydrogel using SF and type III collagen. Figure S1C (Supporting Information) shows the NMR hydrogen spectra of rCol_3_ and rCol_3_MA, and a new acrylic acid proton peak (= CH_2_) clearly appears at 5.3 and 5.6 ppm, which corresponds to the absorption peak of hydrogen on the alkene bond of methylacrylamide, confirming that the modification of rCol_3_ was successful. Figure S1D (Supporting Information) shows the NMR hydrogen spectra of SF and SFMA. Analysis of the NMR results revealed a new acrylic acid proton peak (= CH_2_) at 5.6 and 6.0 ppm, which corresponds to the absorption peak of hydrogen on the alkene bond of methylacrylamide, confirming the successful synthesis of SFMA. Figure S2 (Supporting Information) shows that the network scaffolds of all the hydrogels not only have a certain pore size (90.44±28.63 µm, 76.00±24.76 µm, 63.11±24.92 µm, and 50.47±14.27 µm) but also differ in terms of pore size, pore arrangement, and transport between pores from the local magnified images. However, they all presented 3D reticular structures. This 3D porous network structure can ensure that it has a larger surface area. Overall, these characteristics make the hydrogel structure suitable for loading LNPs and can provide a good barrier and drug delivery system as a dressing for the treatment of diabetic wounds. In addition, as the concentration of rCol_3_MA increased, the network structure of the hydrogel became denser, which was more conducive to cell adhesion, growth and division and more conducive to the transmission of nutrients and oxygen.

### 3.2 Physicochemical Characterization of SFMA/rColMA Hydrogels Loaded with Responsive LNPs

**Figure 2:**
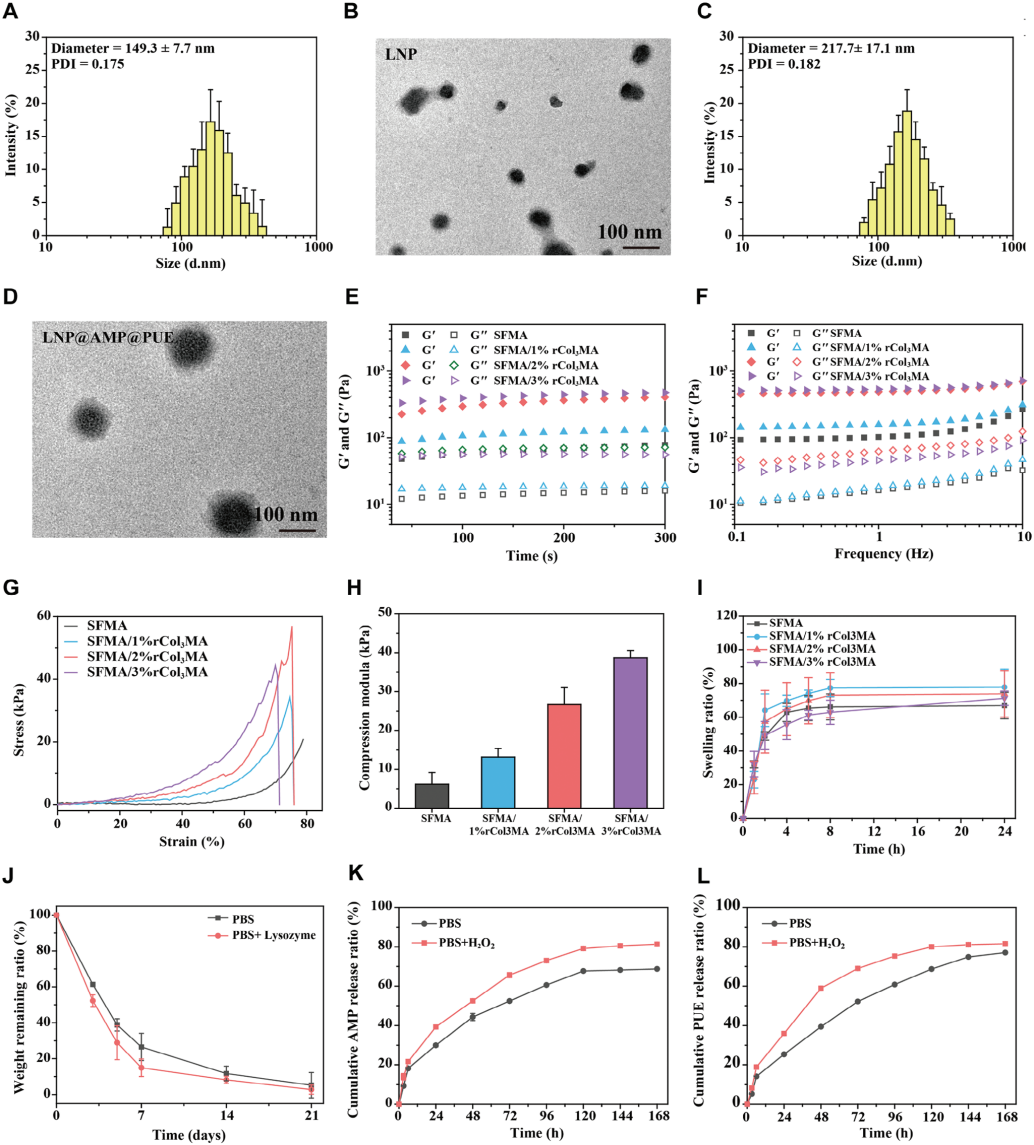
Characterization of SFMA/rColMA hydrogels loaded with ROS‐responsive LNPs. A) Particle size distribution of the LNPs. B) TEM images of the LNPs. Scale bar = 100 nm. C) Particle size distribution of LNP@AMP@PUE. D) TEM images of LNP@AMP@PUE. Scale bar = 100 nm. E) Rheological time‐scan curve of the hydrogel. F) Rheological frequency scanning curve of the hydrogel. G) Stress‒strain curves of the hydrogel. H) Compression module statistics under the maximum strain (*n* = 3). I) Swelling curve of the hydrogel (*n* = 3). J) Degradation curves of the 10% SFMA/2% rCol3MA/LNP@AMP@PUE hydrogel in PBS and enzyme environments (*n* = 3). K) Release rate of AMP in 10%SFMA/2%rCol_3_MA/LNP@AMP@PUE hydrogel. L) Release rate of PUE in 10%SFMA/2%rCol_3_MA/LNP@AMP@PUE hydrogel.

As shown in **Figure** **2**A, the hydrated particle size of the liposomes was 149.3±7.7 nm, and the PDI value was 0.175. TEM images clearly revealed that the liposome, a large single‐compartment liposome resembling a round or oval shape, had a double‐layered structure (Figure 2B). After loading PUE and AMP, the hydrated particle size of the liposomes (LNP@AMP@PUE) was 217.7±17.1 nm, and the PDI value was 0.182 (Figure 2C). After drug loading, the dispersion of liposomes was not affected, and the hydrated particle size was slightly larger. TEM images revealed that the structure of the liposomes was not damaged and that the volume increased (Figure 2D).

The storage modulus (G') and loss modulus (G'') of the hydrogel were characterized by rheology.^[^ ^38^ ^]^ Figure 2E shows that the storage modulus G' was much greater than the dissipation modulus G'' initially, and the modulus no longer changed with time, indicating that the hydrogel had fully formed. In addition, the G' value of the hydrogel also significantly improved with increasing mass fraction of rCol_3_MA, indicating that the strength of the hydrogel increased and that the hydrogel containing 3% rCol_3_MA had the highest storage modulus. Figure 2F shows that G' and G'' basically did not change substantially with increasing frequency when the frequency was varied from 0.1 to 10 Hz, indicating that the linear viscoelastic region of the SFMA/rCol3MA hydrogel was between 0.1 and 10 Hz. The stress‒strain curve of the hydrogel is shown in Figure 2G, which reveals that the trend of stress value variation was consistent with the trend of G' variation measured via rheology. As the mass fraction of rCol_3_MA increased, the maximum stress value (compressive strength) also increased, but the strain value at fracture decreased, indicating that the hydrogel had increased mechanical strength. After calculating the maximum compression strength of the hydrogel when it was broken, we found that the maximum compression strength of SFMA/3%rCol_3_MA was ≈40 kPa, and the compression strength decreased with decreasing rCol_3_MA (Figure 2H).

Figure 2I shows the swelling performance test results of the hydrogel materials. The figure shows that the degree of swelling of all the hydrogels increased with increasing time, and the swelling rates of the four hydrogels were between 50% and 80%. With increasing rCol_3_MA concentration, the swelling rate of the hydrogel decreased somewhat, which was due to the increased cross‐link density, resulting in closer binding between molecular chains and lower water absorption. However, as a material for wound repair, a softer matrix and a higher water absorption rate are more conducive to tissue regeneration. In addition to being a component that improves the mechanical properties of hydrogels, recombinant type III collagen also has biological effects that promote tissue regeneration. Considering the physical and chemical properties and possible biological effects of the hydrogel, we selected SFMA/2% rCol3MA for subsequent experiments.

The degradation performance of the 10%SFMA/2%rCol_3_MA/LNP@AMP@PUE hydrogel (referred to as the SFMA/rColMA/LNP@AMP@PUE hydrogel) was then tested in PBS containing lysozyme and PBS alone. Figure 2J shows the degradation results of the hydrogel at different time points after 21 days of degradation under these two conditions. The weight loss curve of the hydrogel revealed that the hydrogel had a high degradation rate within the initial 7 days of degradation, after which degradation slowed. At 21 days of degradation, the hydrogel with the enzyme was completely degraded, while the hydrogel without the enzyme was degraded to more than 90%. This indicated that the hydrogel material had good biodegradability. Figure 2K,L show the cumulative release curves of AMP and PUE from the SFMA/rColMA/LNP@AMP@PUE hydrogel in a PBS environment and a hydrogen peroxide environment, respectively. Figure S3 (Supporting Information) shows the cumulative release curves of AMP and PUE from LNP@AMP@PUE in both environments. The drug release rate in the H_2_O_2_ environment was greater than that in the absence of H_2_O_2_, and the final cumulative release was also greater, which fully reflects the ROS responsiveness of the carrier.

### 3.3 Evaluation of Hydrogel Biocompatibility and In Vitro Antimicrobial Effects

**Figure 3:**
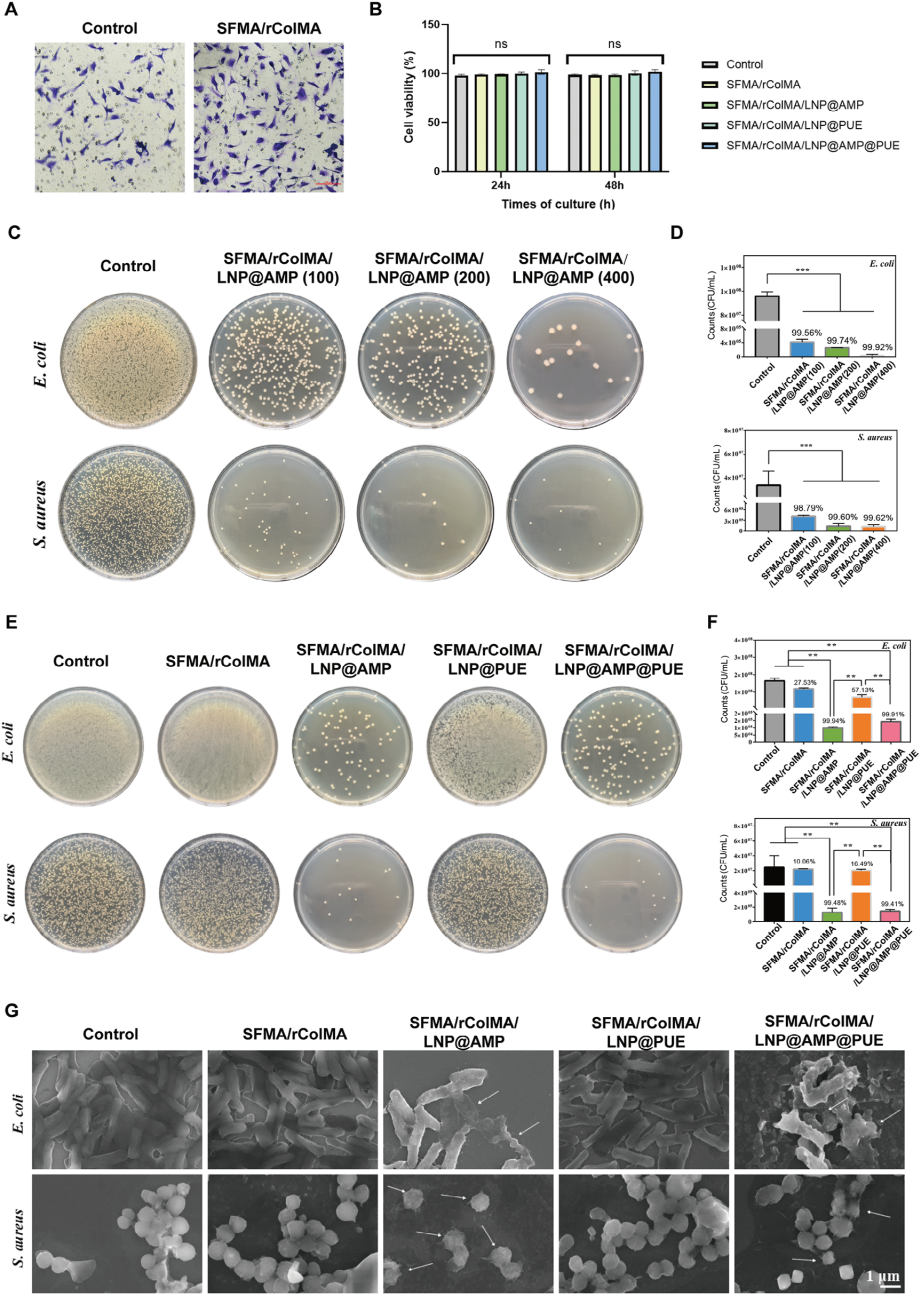
Evaluation of cytocompatibility and antibacterial capacity in vitro. A) The migration ability of fibroblasts in different groups was measured via a Transwell assay (*n* = 3). B) L929 cell viability after treatment with different hydrogels for 24 and 48 h (*n* = 3). C,D): Images and statistics of surviving bacterial clones after treatment with SFMA/rColMA hydrogels containing various concentrations of LNP@AMP (100, 200, or 400 µg mL^−1^) liposomes (*n* = 3). E), F): Images and statistics of surviving bacterial clones after treatment with different hydrogels (*n* = 3). G) SEM images of *E. coli and S. aureus* after different hydrogel treatments (white arrows indicate bacteria that ruptured after injury). Scale bar = 1 µm. ns indicates no significance, ^**^ indicates *p* < 0.01, ^***^ indicates *p* < 0.005.

Given the role of L929 cells in regulating the extracellular matrix, collagen production, and wound contraction,^[^ ^39^ ^]^ L929 cells were used to examine the cytocompatibility of the hydrogels. The results in **Figure** **3**A show that the SFMA/rColMA hydrogel could significantly increase the migration of fibroblasts, which is consistent with previous reports demonstrating that type III collagen can guide fibroblast migration.^[^ ^40^ ^]^ Type III collagen has various biological functions, including providing structural support and regulating cell physiological functions.^[^ ^40^ ^]^ The different hydrogel systems based on SFMA/rColMA also resulted in good cell viability (Figure 3B). These data demonstrated the excellent biocompatibility of our prepared hydrogels, as evidenced by the absence of any noticeable cytotoxic effects.

Diabetic wounds are easily infected by bacteria due to their high‐sugar environment.^[^ ^41^ ^]^ Therefore, a hydrogel with intrinsic antimicrobial capability is desirable. The antibacterial activity of the hydrogel against *E. coli and S. aureus* was evaluated via plate counting. Figure 3C,D shows the antibacterial effects of the SFMA/rColMA hydrogels containing different concentrations of LNP@AMP liposomes against *E. coli and S. aureus*. The antibacterial effect was correlated with the concentration of LNP@AMP, and the higher the concentration, the better was the antibacterial effect. When the concentration of LNP@AMP increased from 100 to 400 µg mL^−1^, the rates against *E. coli* were 99.56%, 99.74%, and 99.92%, and those against *S. aureus* were 98.79%, 99.60%, and 99.62%, respectively. The hydrogels containing LNP@AMP all had significant antimicrobial effects. Considering that the concentration of AMP at 200 µg mL^−1^ could produce a sufficient antibacterial effect and to avoid high concentrations of cell damage, we performed subsequent experiments with 200 µg mL^−1^ LNP@AMP, denoted as SFMA/rColMA/LNP@AMP. The killing effect of hydrogels containing different liposomes on bacteria was subsequently studied. SFMA/rColMA/LNP@AMP and SFMA/rColMA/LNP@AMP@PUE containing AMP showed good antibacterial effects, and the antibacterial effects on *E. coli and S. aureus* reached more than 99% (Figure 3E,F). After further observation of the bacterial morphology (Figure 3G), we found that the bacteria treated with AMP were wrinkled and ruptured (indicated by white arrows). The remaining groups were complete and saturated. These results indicated that AMP exerted a good antibacterial effect, whereas the SFMA/rColMA/LNP@AMP@PUE hydrogel also had good antibacterial activity. The main mechanism of the antibacterial action of AMP is not only limited to its membrane‐disrupting effect but also involves the modulation of intracellular targets involved in DNA, RNA, or protein synthesis.^[^ ^10^, ^11^, ^12^ ^]^

### 3.4 Analysis of the Associations between Diabetic Wounds and PUE Targets

Previous studies have demonstrated the anti‐inflammatory and antioxidative stress properties of PUE.^[^ ^42^, ^43^ ^]^ However, few studies have investigated the potential relationship between PUE and the wound microenvironment. Therefore, we initially employed bioinformatics to elucidate this association.

**Figure 4:**
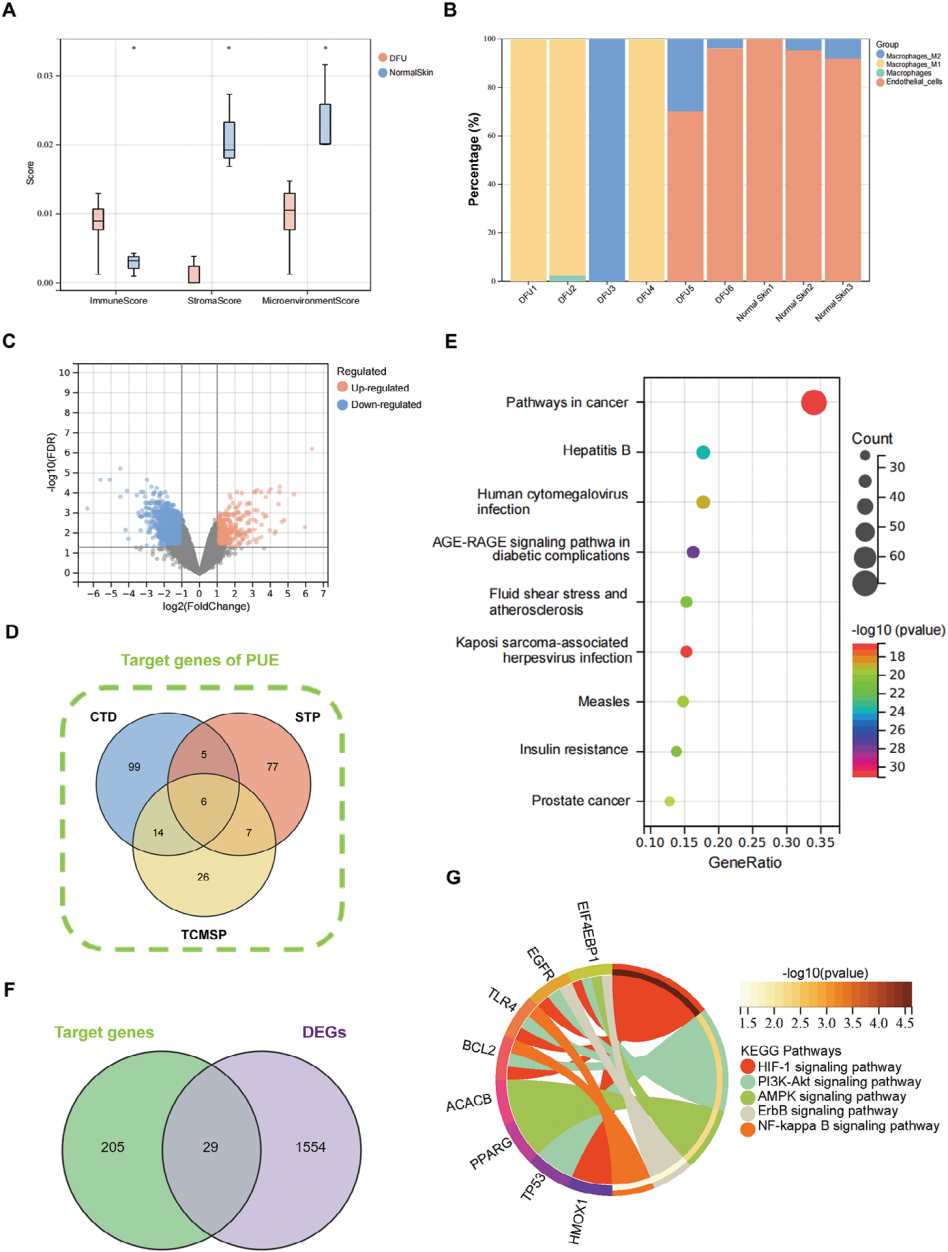
Analysis of the associations between diabetic wounds and PUE targets via network pharmacology. A) Distribution of the immune, stroma, and microenvironment scores of DFUs and normal skin. B) Fractions of macrophages and endothelial cells from DFUs and normal skin tissues. C) Volcano plot of DEGs from the GSE80178 dataset. D) Summary of target genes from different databases. E) Results of the KEGG pathway enrichment analysis results of target genes. F) Venn diagram of common genes. G) KEGG pathway enrichment analysis results of common genes. ^*^ indicates *p* < 0.05.

To reveal the diabetic wound microenvironment, we used the xCell algorithm to calculate the microenvironment score and perform a cellular component analysis of the dataset. As shown in **Figure** **4**A, the microenvironment score and stroma score of diabetic wounds were significantly lower than those of normal skin, whereas the immune score was significantly greater than that of normal skin. Given the important role of macrophages and vascular endothelial cells in wound healing, we focused on the corresponding results of the component analysis. As shown in Figure 4B, we found that the infiltration of macrophages in diabetic wounds was significantly increased, and M1 macrophages were the main type, while the number of endothelial cells was significantly lower than that in the normal group. These results were consistent with the trend of the scoring results in the previous step. A differential analysis of the transcriptome revealed that a total of 1583 genes presented significant expression changes, with 356 genes being upregulated and 1227 genes being downregulated (Figure 4C).

To further explore the potential of PUE, we identified 124, 53, and 95 targets from the CTD, TCMSP, and STP databases, respectively, to acquire the corresponding targets associated with PUE (Table S4, Supporting Information). After removing duplicates, a total of 234 targets were obtained (Figure 4D). These 234 target genes were associated mainly with cancer, infectious diseases, diabetes mellitus, and other diseases (Figure 4E). We then used a Venn diagram to show the intersection of diabetes wound‐related DEGs and PUE targets, obtaining 29 genes for subsequent bioinformatics analysis (Figure 4F). According to the results shown in the figure, PUE is involved mainly in the HIF‐1 signaling pathway, the PI3K‐Akt signaling pathway, and other pathways (Figure 4G). These pathways are relevant to the regulation of vascular endothelial cell function and macrophage polarization.^[^ ^44^, ^45^ ^]^ Therefore, we hypothesized that PUE might ameliorate wound oxidative stress and inflammation dysregulation by regulating these processes, thereby improving the microenvironment of diabetic wounds.

### 3.5 Angiogenic Effects of the SFMA/rColMA/LNP@AMP@PUE Hydrogel Under ROS Imbalance

**Figure 5:**
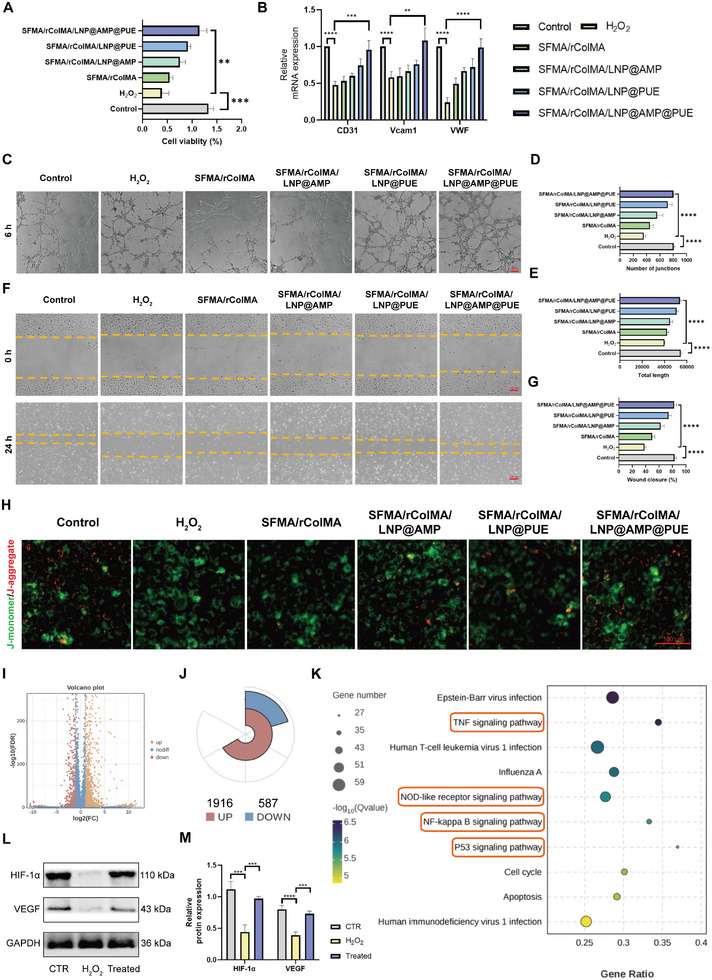
Effect of the SFMA/rColMA/LNP@AMP@PUE hydrogel on angiogenesis in the ROS microenvironment. A) Viability of HUVECs incubated with different hydrogels (*n* = 3). B) qRT‒PCR assay of the gene expression of angiogenesis‐related genes (CD31, Vcam1 and VWF) in HUVECs treated with different hydrogels (*n* = 3). C) Optical images of tube formation by HUVECs treated with different hydrogels. Scale bar = 100 µm. D), E): Quantification of the number of junctions and total length in each group (*n* = 3). F) Scratch assay images of HUVECs treated with different hydrogels. Scale bar = 100 µm. G) Quantification of the area change was performed (*n* = 3). H) Representative image and analysis of the mitochondrial membrane potential. JC‐1 was used to stain mitochondria with a strong membrane potential (red) and mitochondria with a weak membrane potential (green). Scale bar = 100 µm. I) Volcano map of the genes differentially expressed between the H_2_O_2_ group and the hydrogel‐treated group. J) Statistical plot of the differentially expressed genes. K) Pathway enrichment factor map of differentially expressed genes. L) Western blot analysis of HIF1‐α and VEGF expression in HUVECs after treatment with H_2_O_2_ and the SFMA/rColMA/LNP@AMP@PUE hydrogel. M) Quantitative analysis of HIF1‐α and VEGF expression via western blotting (*n* = 3). ^**^ indicates *p* < 0.01, ^***^ indicates *p* < 0.005, ^****^ indicates *p* < 0.001.

The characteristic manifestation of chronic diabetic wounds is vascular function impairment associated with ROS imbalance. To simulate oxidative stress and the ROS microenvironment, we conducted an experiment using H_2_O_2_ at a concentration of 200×10^−6^ m in vitro.^[^ ^46^ ^]^ The untreated HUVEC group was used as a control group. The viability of HUVECs in the ROS microenvironment was initially assessed (**Figure** **5**A). After 3 days of continuous culture, the viability of HUVECs was significantly inhibited by oxidative stress damage. However, all other treatment groups exhibited varying degrees of cell viability restoration, with the SFMA/rColMA/LNP@AMP@PUE hydrogel group demonstrating the most pronounced effect.

The process of wound healing involves the secretion of various vasoactive substances by endothelial cells, which synergistically promote vascular formation through their cytokine actions.^[^ ^47^ ^]^ qRT‒PCR analysis revealed significant upregulation of the expression of angiogenesis‐related genes, including CD31, vascular cell adhesion molecule 1 (Vcam1), and von Willebrand factor (VWF), in the SFMA/rColMA/LNP@AMP@PUE treatment group compared with the H_2_O_2_ group (Figure 5B). Neovascularization facilitates wound healing by providing a continuous supply of oxygen and nutrients to the injured area.^[^ ^48^ ^]^ The migration, proliferation, and tube formation of vascular endothelial cells are crucial steps in achieving therapeutic efficacy. The tube‐forming ability decreased significantly under the influence of H_2_O_2_, as shown in Figure 5C–E. However, when treated with the SFMA/rColMA/LNP@AMP@PUE hydrogel, the quantity of HUVECs that formed tubes increased noticeably, and more distinct tubular structures were observed. Additionally, the migration ability of the SFMA/rColMA/LIP@AMP@PUE hydrogel group was significantly greater than that of the other groups (Figure 5F,G). The continuous presence of high glucose in the environment has been reported to induce metabolic stress and mitochondrial damage, among other effects.^[^ ^49^, ^50^, ^51^ ^]^ Additionally, vascular damage associated with dysregulated ROS in diabetic wounds is closely linked to impaired mitochondrial dynamics.^[^ ^49^, ^50^, ^51^ ^]^ The results of the JC‐1 test revealed that H_2_O_2_ treatment significantly decreased the intracellular mitochondrial membrane potential (φM) in HUVECs. However, when treated with the SFMA/rColMA/LNP@AMP@PUE hydrogel, φM was preserved and exhibited a superior effect (Figure 5H).

To further explore the potential mechanism by which the SFMA/rColMA/LNP@AMP@PUE hydrogel promoted vascularization, we performed transcriptome sequencing of HUVECs. Compared with the H_2_O_2_‐treated samples, the samples treated with the SFMA/rColMA/LNP@AMP@PUE hydrogel presented obvious changes at the gene transcription level, and a total of 2503 DEGs were identified, of which 1916 genes were upregulated and 587 genes were downregulated (Figure 5I,J). DEGs were classified via KEGG pathway analysis. Compared with the H2O2‐treated group, the SFMA/rColMA/LNP@AMP@PUE hydrogel significantly affected multiple signaling pathways in HUVECs. Considering the important therapeutic effects of PUE in our study and the network pharmacology analysis results, the NF‐kappa B signaling pathway in particular attracted our attention (Figure 5K). NF‐kappa B is an important direct regulator of HIF‐1α expression.^[^ ^52^ ^]^ The complex formed by HIF‐1α and p300, through binding to hypoxia response elements, can upregulate the expression of several angiogenic genes, such as VEGF.^[^ ^53^, ^54^ ^]^ The VEGF pathway is a major regulator of angiogenesis and a key regulator of vascular development.^[^ ^55^ ^]^ Vascular dysfunction strongly hinders the healing process of diabetic wounds while activating the expression of HIF‐1α, and VEGF can promote wound healing.^[^ ^56^ ^]^ To investigate whether the SFMA/rColMA/LNP@AMP@PUE hydrogel could promote angiogenesis through the HIF‐1α pathway, we performed western blot experiments. Under oxidative stress, the HIF‐1α pathway was significantly inhibited, but the SFMA/rColMA/LNP@AMP@PUE hydrogel significantly activated the HIF‐1α pathway, thereby restoring the expression of VEGF (Figure 5L,M).

Furthermore, the other treatment groups exhibited partial recovery in the aforementioned experiments, with the PUE‐loaded hydrogel group demonstrating relatively prominent advantages, which could be attributed to the antioxidative stress properties of PUE.^[^ ^42^, ^43^ ^]^ Collectively, these findings indicated that the SFMA/rColMA/LNP@AMP@PUE hydrogel was efficacious in promoting angiogenesis within the ROS microenvironment.

### 3.6 Anti‐Inflammatory Capacity of the SFMA/rColMA/LNP@AMP@PUE Hydrogel under ROS Imbalance

The occurrence of high levels of oxidative stress‐mediated by ROS in diabetic wounds triggers metabolic changes in glycolysis and activates inflammatory pathways related to monocyte‐macrophages, ultimately resulting in impaired immune function in diabetic wounds.^[^ ^57^, ^58^, ^59^ ^]^ Considering the crucial role of macrophages in wound healing, particularly M2 macrophages, which promote tissue repair and have anti‐inflammatory properties, while M1 macrophages are associated with early inflammation during wound healing, our aim was to investigate the impact of the SFMA/rColMA/LNP@AMP@PUE hydrogel on macrophage polarization in a simulated ROS microenvironment in vitro.

**Figure 6:**
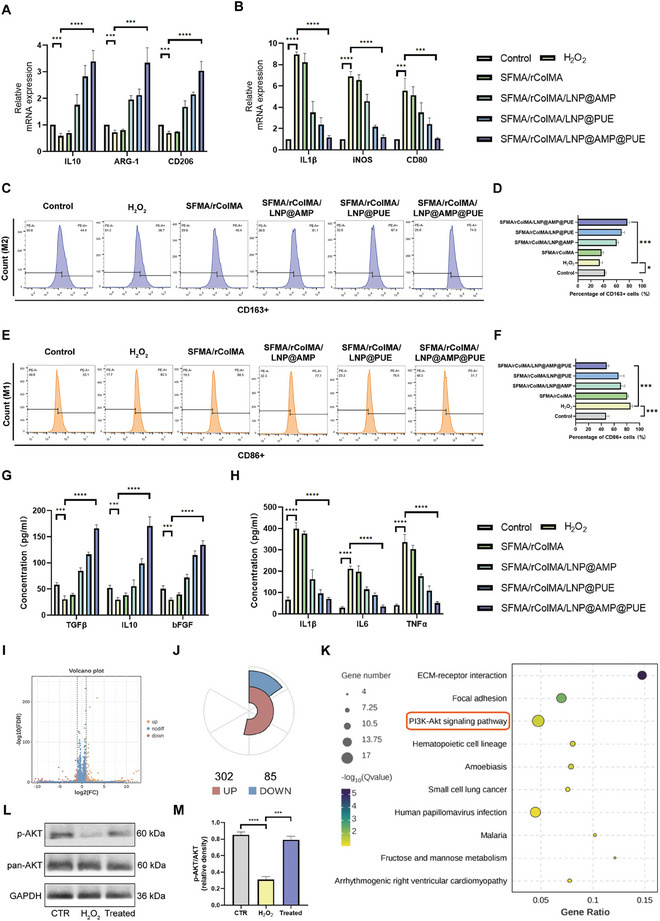
Effect of the SFMA/rColMA/LNP@AMP@PUE hydrogel on the polarization of macrophages in the ROS microenvironment. A), B): qRT‒PCR analysis of the expression of typical M2 markers (IL‐10, ARG‐1, and CD206) and M1 markers (IL‐1β, iNOS, and CD80) in PMA‐pretreated THP‐1 cells treated with different hydrogels (*n* = 3). C), D), E), F): The flow cytometry results of PMA‐pretreated THP‐1 cells treated with different hydrogels were stained with the M1 marker CD86 and the M2 marker CD163 (*n* = 3). G,H): The expression levels of cytokines (TGF‐β, IL10, bFGF, IL‐1β, IL6, and TNF‐α) in PMA‐pretreated THP‐1 cells treated with NG/CMCS and NG/CMCS/HA/SF scaffolds were detected via ELISA (*n* = 3). I) Volcano map of the differentially expressed genes between the H_2_O_2_ group and the hydrogel‐treated group. J) Statistical plot of the differentially expressed genes. K) Pathway enrichment factor map of differentially expressed genes. L) Western blot analysis of AKT and p‐AKT expression in macrophages after treatment with H_2_O_2_ and the SFMA/rColMA/LNP@AMP@PUE hydrogel. M) Quantitative analysis of AKT and p‐AKT expression via western blotting (*n* = 3). ^**^ indicates *p* < 0.01, ^***^ indicates *p* < 0.005, ^****^ indicates *p* < 0.001.

We initially established the ROS microenvironment through the addition of H_2_O_2_ and subsequently performed qRT‒PCR, flow cytometry, and enzyme‒linked immunosorbent assay (ELISA) analyses on the cocultured cells. The expression of anti‐inflammatory markers (CD206, IL10, and ARG‐1) and proinflammatory markers (IL1β, CD80, and iNOS) is shown in **Figure** **6**A,B. Notably, the genes associated with both the M2 phenotype and the M1 phenotype were significantly upregulated in the SFMA/rColMA/LNP@AMP@PUE hydrogel group. The flow cytometry results revealed the distribution of macrophages treated with different hydrogel systems. As shown in Figure 6C,D, the addition of H_2_O_2_ significantly increased the proportion of CD86‐positive THP‐1 (PMA‐treated) cells while reducing the proportion of CD206‐positive cells, indicating predominant polarization toward the M1 macrophage phenotype. Importantly, the introduction of the SFMA/rColMA/LNP@AMP@PUE hydrogel effectively activated the M2 macrophage phenotype in THP‐1 (PMA‐treated) cells and attenuated H_2_O_2_‐induced M1 macrophage polarization (Figure 6E,F). Additionally, ELISA analysis revealed significant upregulation of anti‐inflammatory cytokines (TGFβ, bFGF and IL10) and notable downregulation of proinflammatory cytokines (TNFα, IL‐1β and IL‐6) in the SFMA/rColMA/LNP@AMP@PUE hydrogel group (Figure 6G,H), which was consistent with the above findings.

To further investigate the anti‐inflammatory mechanism of the hydrogel, we also screened the key signaling pathways by which the SFMA/rColMA/LNP@AMP@PUE hydrogel regulated macrophage polarization via transcriptome analysis. Compared with those in the H_2_O_2_‐treated group, the gene transcription levels significantly changed after SFMA/rColMA/LNP@AMP@PUE hydrogel treatment, of which 302 genes were significantly upregulated and 85 genes were significantly downregulated (Figure 6I,J). The results of KEGG analysis revealed that these significantly differentially expressed genes were significantly enriched in the PI3K‐AKT signaling pathway, and the results of network pharmacological analysis also indicated that the PI3K‐AKT signaling pathway was potentially important for PUE in the treatment of diabetic wounds (Figure 6K). The PI3K/AKT pathway is a signaling pathway related to cell proliferation, differentiation, migration; protein synthesis; and the inhibition of the inflammatory response. Elevated levels of phosphorylated PI3K‐AKT can regulate downstream signal transduction, regulate ROS levels, promote M2 macrophage polarization, and remodel the inflammatory microenvironment.^[^ ^60^ ^]^ The western blot results revealed that the PI3K‒AKT pathway in macrophages was significantly inhibited under oxidative stress, which was consistent with previous reports,^[^ ^61^ ^]^ and the PI3K‒AKT pathway was significantly activated by intervention with the SFMA/rColMA/LNP@AMP@PUE hydrogel (Figure 6L,M).

Our findings collectively demonstrated the ability of the SFMA/rColMA/LNP@AMP@PUE hydrogel to effectively suppress the inflammatory phenotype of macrophages while promoting an anti‐inflammatory phenotype. Furthermore, the specific cytokines produced by M2 macrophages possess multifunctional properties crucial for tissue regeneration.^[^ ^62^, ^63^ ^]^ Notably, compared with hydrogels loaded with only AMP, hydrogels loaded with only PUE exhibited superior efficacy in promoting macrophage polarization toward the M2 phenotype.

### 3.7 In Vivo Wound Healing Effects of the SFMA/rColMA/LNP@AMP@PUE Hydrogel

**Figure 7:**
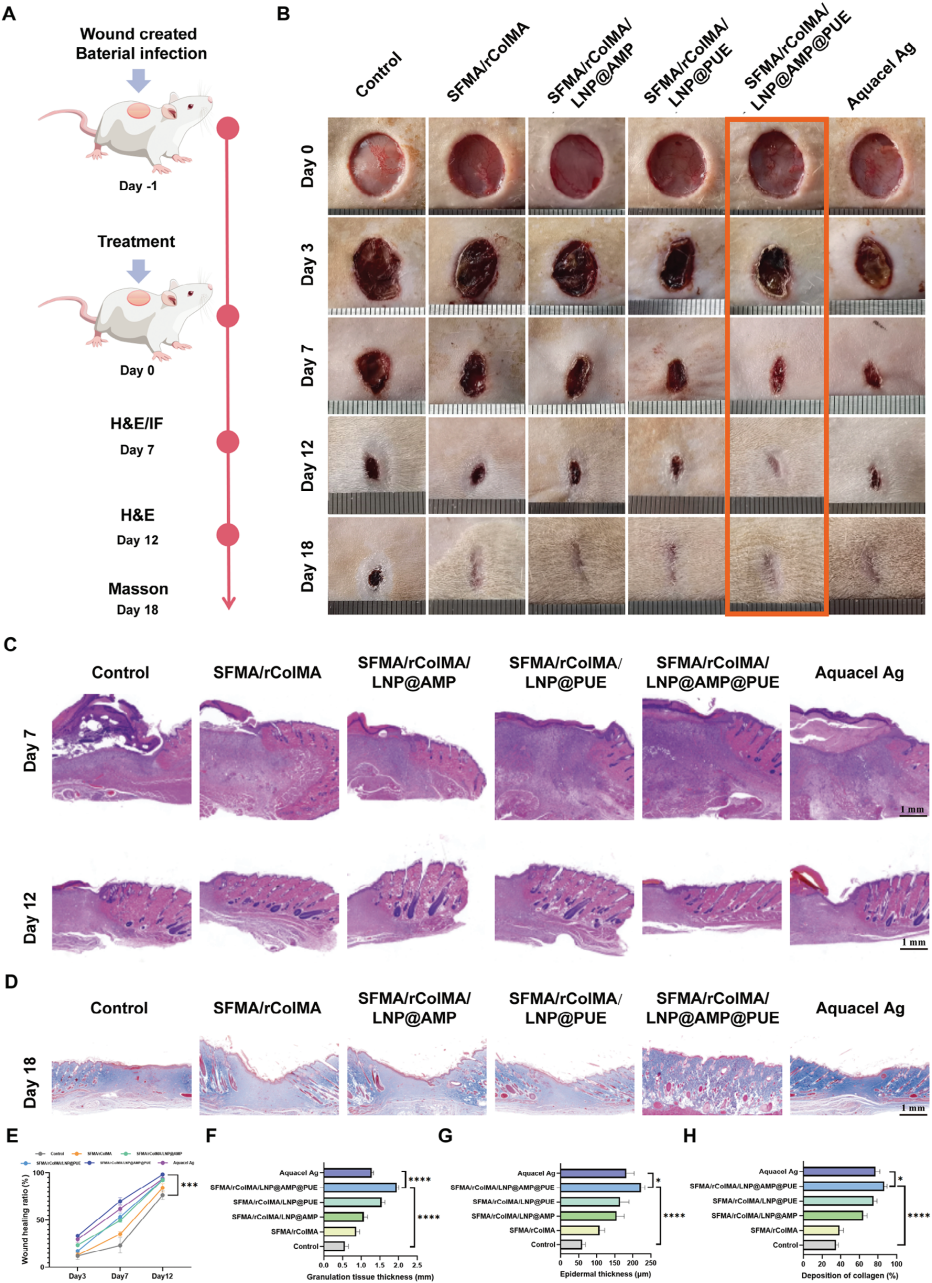
Assessment of wound healing in infected diabetic patients treated with different hydrogels. A) Treatment schedule for infected diabetic wounds treated with different hydrogels. B) Representative images of infected diabetic wounds at different times. C) HE staining of wound sections from all groups on Days 7 and 12. Scale bar = 1 mm. D) Masson staining images of wounds in all groups on Day 18. E) Quantification of the wound healing rate after treatment (*n* = 3). Scale bar = 1 mm. F) Statistics of granulation tissue thickness on Day 7 (*n* = 3). G) Collagen accumulation on Day 12, as determined via Masson's trichrome staining (*n* = 3). H) Statistical analysis of epidermal thickness on Day 18 (*n* = 3). ^*^ indicates *p* < 0.05, ^***^ indicates *p* < 0.005, and ^****^ indicates *p* < 0.001.

The wound healing efficacy of the SFMA/rColMA/LNP@AMP@PUE hydrogel was assessed in an infectious diabetic skin wound model. **Figure** **7**A illustrates the evaluation strategy for in vivo treatment.

The wounds in the experimental group were treated with Aquacel Ag, a commercially available dressing, or hydrogels from various systems, whereas the untreated wounds served as the control group. Macroscopic photographs of the wounds in each group are presented in Figure 7B. Compared with the Aquacel Ag group and the blank group, the SFMA/rColMA/LNP@AMP@PUE hydrogel group demonstrated significantly enhanced wound closure (Figure 7E). By Day 12, the wounds treated with the SFMA/rColMA/LNP@AMP@PUE hydrogel exhibited substantial healing. The healing rates in the SFMA/rColMA/LNP@AMP and SFMA/rColMA/LNP@PUE hydrogel groups were significantly faster than those in the blank and SFMA/rColMA groups. However, these effects were still slower than those observed in the SFMA/rColMA/LNP@AMP@PUE hydrogel group, indicating that the combined application of AMP and PUE in the hydrogel effectively promoted diabetic infected wound healing that surpassed the healing rate with single applications.

As mentioned earlier, wound healing is a complex and continuous process.^[^ ^64^ ^]^ We therefore further evaluated the histological status during wound healing by histological analysis on Days 7, 12, and 18. Figure 7C shows the results of HE staining of the skin tissues. Compared with the other groups, the SFMA/rColMA/LNP@AMP@PUE hydrogel group presented significantly more granulation tissue on Day 7 (Figure 7F). On Day 12, all the groups presented newly formed epidermis, but the SFMA/rColMA/LNP@AMP@PUE hydrogel group presented a relatively thicker and more natural and mature epidermal layer than did the other groups (Figure 7G). Collagen deposition in the wound healing zone was evaluated via Masson's trichrome staining on Day 18, revealing an increase in collagen content across all the treatment groups (Figure 7D). Compared with the blank and Aquacel Ag groups, the SFMA/rColMA/LNP@AMP@PUE hydrogel group presented greater collagen deposition on Day 18 (Figure 7H). The SFMA/rColMA/LNP@AMP@PUE hydrogel group exhibited a dense, thick and well‐organized structure with abundant hair follicles. In conclusion, the SFMA/rColMA/LNP@AMP@PUE hydrogel accelerated the formation of epidermal and dermal tissue in infected diabetic wounds, thereby achieving satisfactory healing efficacy.

### 3.8 In Vivo Antibacterial Properties, Anti‐Inflammatory Properties, and Vascularization Capacity of the SFMA/rColMA/LNP@AMP@PUE Hydrogel

The microenvironment of chronic diabetic wounds is characterized mainly by bacterial infection, inflammatory disorders, cell proliferation inhibition, and angiogenesis disorders, among others. To verify whether the SFMA/rColMA/LNP@AMP@PUE hydrogel could promote healing by regulating the wound microenvironment, further tests were conducted as follows.

**Figure 8:**
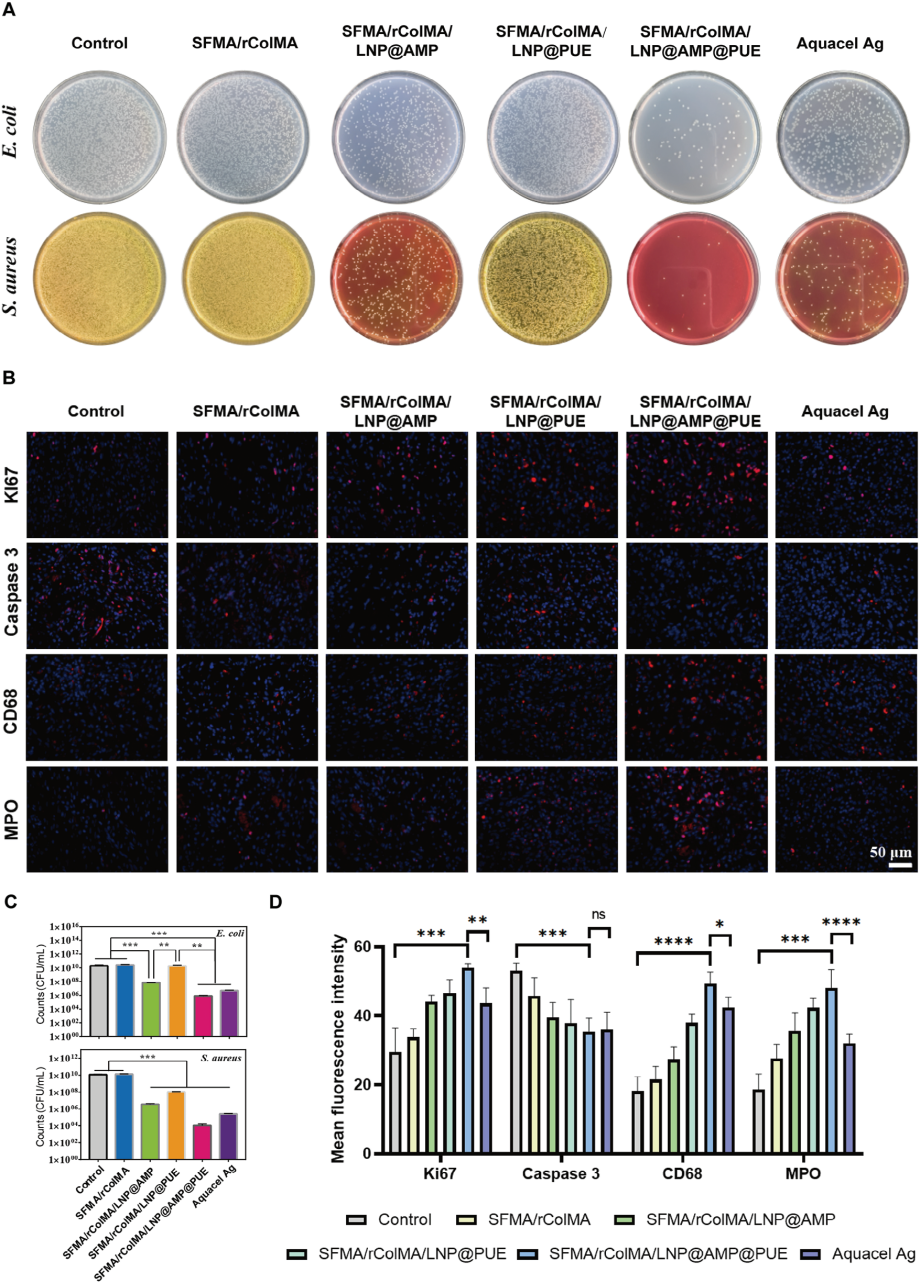
Evaluation of the antimicrobial ability and microenvironment regulation of the hydrogels in vivo. A,C): Images and statistics of surviving bacterial clones on Day 3 in vivo (*n* = 3). B), D): Immunofluorescence staining and statistical analysis of Ki67, Caspase 3, MPO, and CD68 expression in the wound tissues on Day 7 (*n* = 3). Scale bar = 50 µm. ^*^ indicates *p* < 0.05, ^***^ indicates *p* < 0.005, and ^****^ indicates *p* < 0.001.

The antimicrobial properties of the prepared hydrogels were further evaluated in vivo by collecting bacteria from the wound area after 3 days of treatment, in order to assess the bacterial count at the wound site. Our analyses revealed that wounds treated with hydrogel loaded with AMP exhibited significantly reduced colony formation compared with those in the other groups (**Figure** **8**A,C). Additionally, the hydrogel loaded solely with PUE also demonstrated partial antibacterial efficacy, while the SFMA/rColMA/LNP@AMP@PUE hydrogel displayed the highest level of antibacterial effectiveness. These findings suggested that hydrogels loaded with AMP could exert high bactericidal activity, while the co‐loading of PUE would enhance their in vivo bacterial elimination efficacy. This result further demonstrated the antimicrobial properties of the SFMA/rColMA/LNP@AMP@PUE hydrogel.

To further assess the changes in the wound microenvironment, Day 7 wound tissue was specifically chosen for immunofluorescence staining to facilitate evaluation (Figure 8B,D). The cell proliferation antigen Ki‐67 is consistently expressed during mammalian cell division and serves as an indispensable protein for cellular proliferation, rendering it widely employed as a marker to evaluate cell proliferation.^[^ ^65^, ^66^ ^]^ Immunostaining for Ki‐67 revealed a significant increase in overall wound proliferation subsequent to treatment with the SFMA/rColMA/LNP@AMP@PUE hydrogel. Moreover, the expression of the apoptosis‐related protein caspase‐3 was significantly downregulated upon treatment with the SFMA/rColMA/LNP@AMP@PUE hydrogel. The cysteine protease caspase‐3 is intricately involved in cellular apoptosis, playing a pivotal role in embryonic development and maintaining cell homeostasis by degrading specific proteins that undergo apoptotic processes.^[^ ^67^ ^]^ In conclusion, the intervention of the SFMA/rColMA/LNP@AMP@PUE hydrogel actively mobilized the vitality of cells in wounds in patients with infectious diabetes.

**Figure 9:**
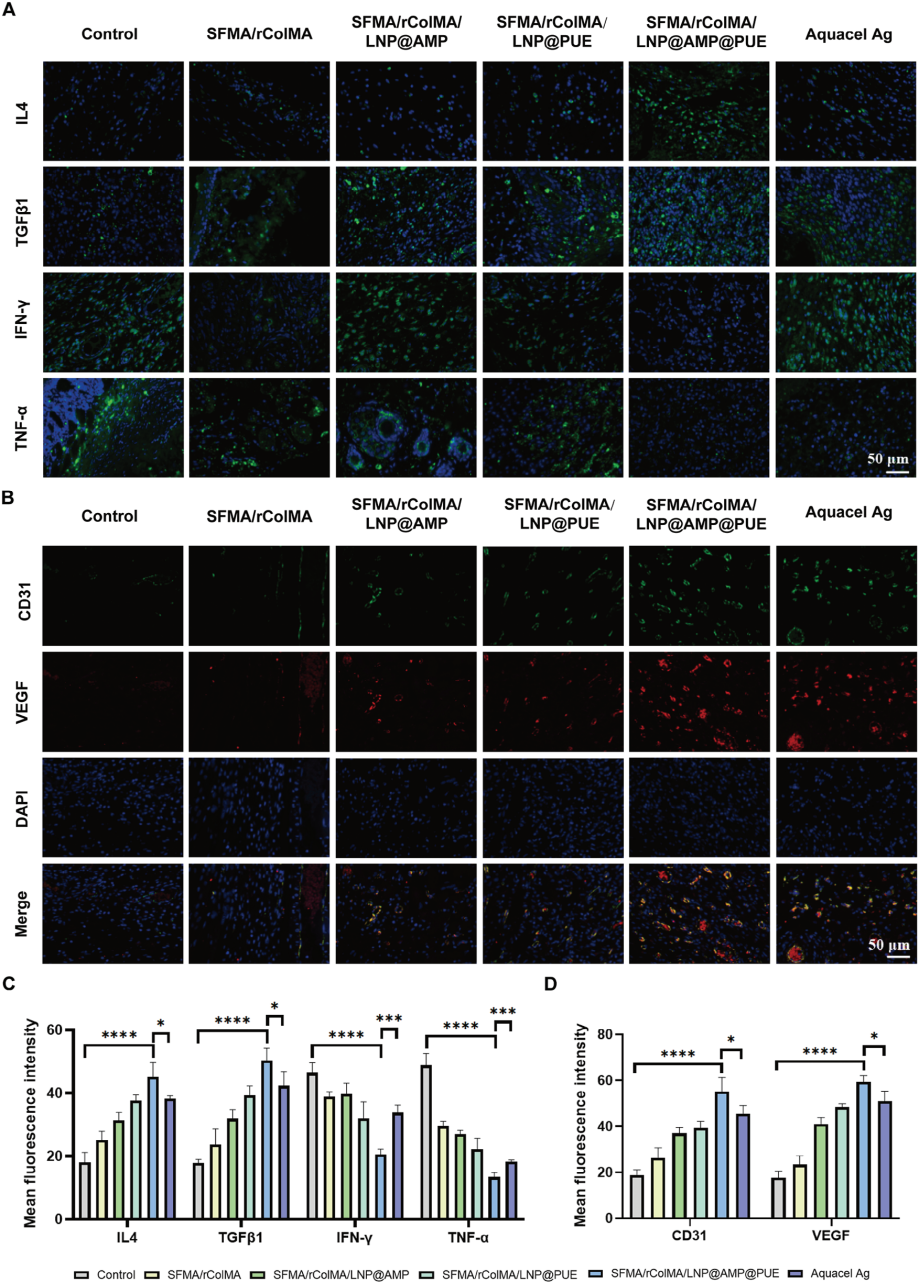
In vivo anti‐inflammatory and vascularization capacity of the hydrogels. A,C): Immunofluorescence images and statistics of IL4, TGFβ1, IFN‐γ, and TNF‐α in the wounds on Day 7 after different treatments (*n* = 3). Scale bar = 50 µm. B,D): Immunofluorescence images and statistics of CD31 and VEGF expression in the wounds on Day 7 after different treatments (*n* = 3). Scale bar = 50 µm. ^*^ indicates *p* < 0.05, ^***^ indicates *p* < 0.005, and ^****^ indicates *p* < 0.001.

The transition from the inflammatory phase to the proliferative phase represents a critical regulatory point in the wound healing process, and persistent inflammation is among the most prominent characteristics observed in chronic diabetic wounds.^[^ ^68^ ^]^ By detecting immune cell markers, we observed significant infiltration of MPO+‐labeled neutrophils and CD68+‐labeled macrophages in the SFMA/rColMA/LNP@AMP@PUE hydrogel group. Additionally, there was notable upregulation of the expression of anti‐inflammatory‐related IL4 and TGFβ1, while the expression of proinflammatory‐related IFN‐γ and TNF‐α was significantly downregulated (**Figure** **9**A,C). Compared with the control group, the SFMA/rColMA/LNP@AMP@PUE hydrogel actively coordinated with the immune status of the wound, effectively mitigated the inflammatory response, and significantly enhanced phenotype transformation toward an anti‐inflammatory and pro‐healing state, surpassing the other treatment groups by a considerable margin.

The local vasculature plays a crucial role in supplying wounds with oxygen, nutrients, and key growth factors necessary for promoting local regeneration.^[^ ^69^, ^70^ ^]^ However, inadequate angiogenesis is a common characteristic of diabetic infected wounds, highlighting the importance of neovascularization in tissue repair. To evaluate the hydrogel's vascularization potential, we assessed the expression levels of CD31 and VEGF, two well‐established markers associated with angiogenesis. As shown in Figure 9B,D, the expression of both VEGF and CD31 was significantly increased on Day 7 after the application of the SFMA/rColMA/LNP@AMP@PUE hydrogel compared with that in the control and Aquacel Ag groups.

In summary, the SFMA/rColMA/LNP@AMP@PUE hydrogel clearly enhanced neovascularization while eliminating bacterial infection, stimulating wound viability, and inhibiting inflammatory responses. These results indicated that the SFMA/rColMA/LNP@AMP@PUE hydrogel played a more positive role in the regulation of the wound microenvironment than did simple hydrogels or single‐drug‐loaded hydrogels, reflecting the advantages of high‐potency biological dressings formed by the combination of a controlled release system and multiple drugs, which could significantly improve the therapeutic effect of tissue regeneration.

## 4 Conclusion

In this study, we developed a novel multifunctional hydrogel that could serve as a wound dressing to expedite the healing process of chronic diabetic wounds by mitigating bacterial infection, alleviating oxidative stress and inflammation, and promoting collagen deposition and angiogenesis. This hydrogel is formulated using SFMA and rColMA as base materials combined with ROS‐responsive LNPs loaded with AMP and PUE, thereby conferring favorable physical characteristics to the hydrogel while enabling it to respond effectively to ROS stimuli. Therefore, the development of this multifunctional hydrogel will help improve patient outcomes and quality by promoting rapid healing of diabetic wounds. Moreover, considering that there are other types of chronic nonhealing wounds in clinical practice, this novel hydrogel also has considerable application potential. Collectively, these results suggest that the multifunctional SFMA/rColMA/LNP@AMP@PUE hydrogel holds great promise as a strategy for regulating the diabetic wound microenvironment and may pave the way for further advancements in stimulus‐responsive wound dressings.

## Conflict of Interest

The authors declare no conflict of interest.

## Supporting information



Supporting Information

## Data Availability

The data that support the findings of this study are available from the corresponding author upon reasonable request.
