# Supplementary material for: A ROS‐Responsive Lipid Nanoparticles Release Multifunctional Hydrogel Based on Microenvironment Regulation Promotes Infected Diabetic Wound Healing
Source: Adv Sci (Weinh). 2024 Sep 23;11(43):2403219. doi: 10.1002/advs.202403219 (PMC11578374; doi:10.1002/advs.202403219)
Supplement: Supplementary file 1 — Supporting Information [file ADVS-11-2403219-s001.pdf]

ADVANCED SCIENCE
Open Access

Supporting Information

for *Adv. Sci.*, DOI 10.1002/advs.202403219

A ROS-Responsive Lipid Nanoparticles Release Multifunctional Hydrogel Based on Microenvironment Regulation Promotes Infected Diabetic Wound Healing

Hao Yang, Dongming Lv, Shanqiang Qu, Hailin Xu, Shuting Li, Zhiyong Wang, Xiaoling Cao, Yanchao Rong, Xiaohui Li, Honglin Wu, Yongfei Chen, Jiayuan Zhu, Bing Tang* and Zhicheng Hu**

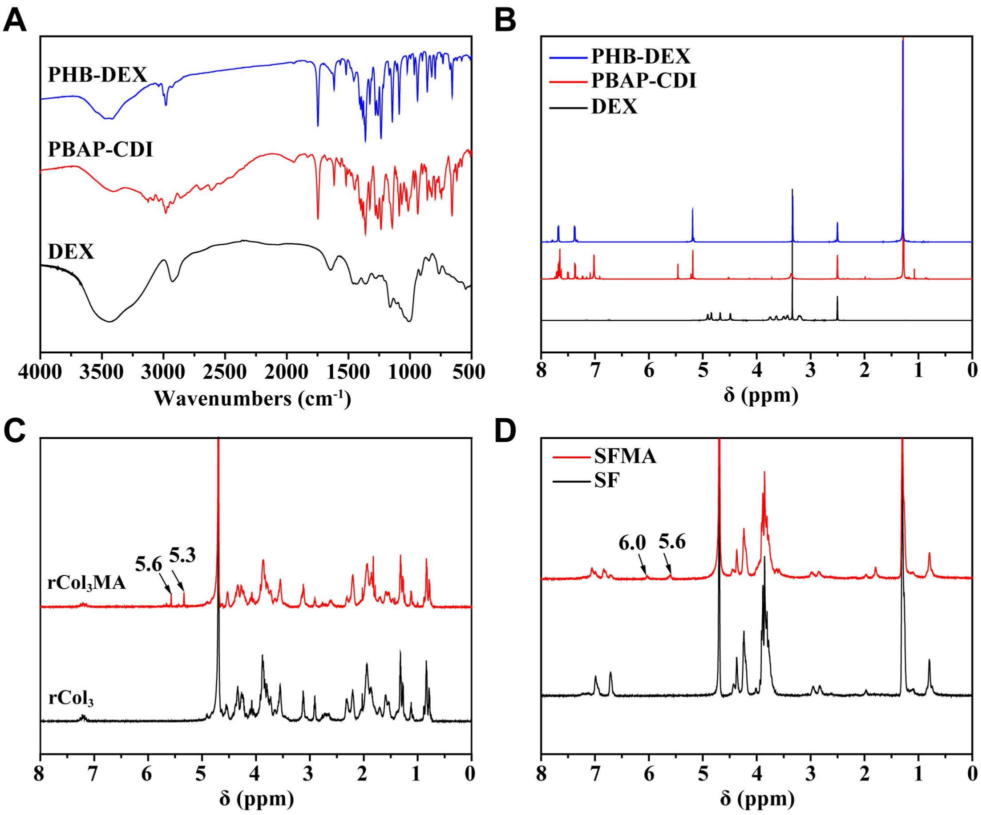

Figure S1. Chemical structure analysis of LNP and hydrogels. A) IR profiles of DEX, PBAP-CDI and PHB-DEX. B) Hydrogen NMR spectra of DEX, PBAP-CDI and PHB-DEX. C) Hydrogen NMR spectra of rCol3, rCol3MA. D) NMR hydrogen spectra of SF and SFMA.

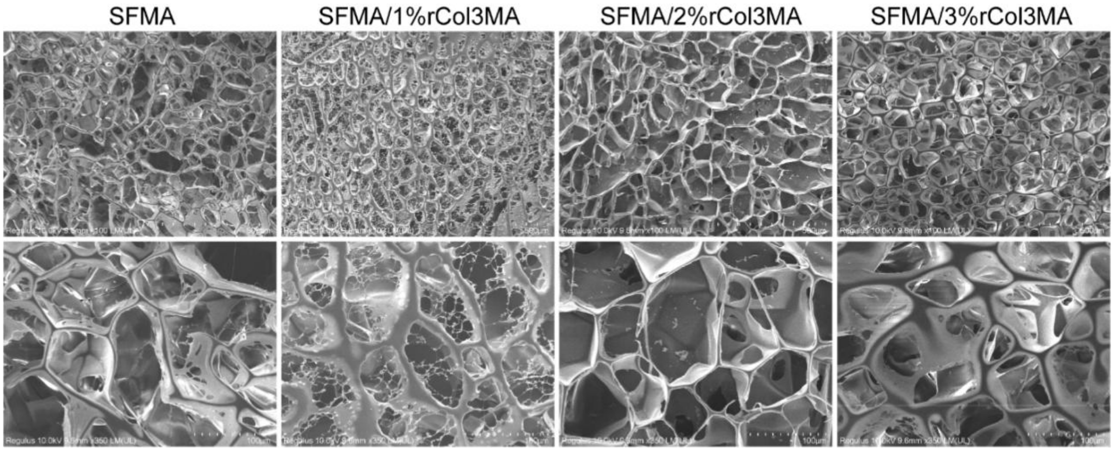

Figure S2. SEM images of different hydrogels.

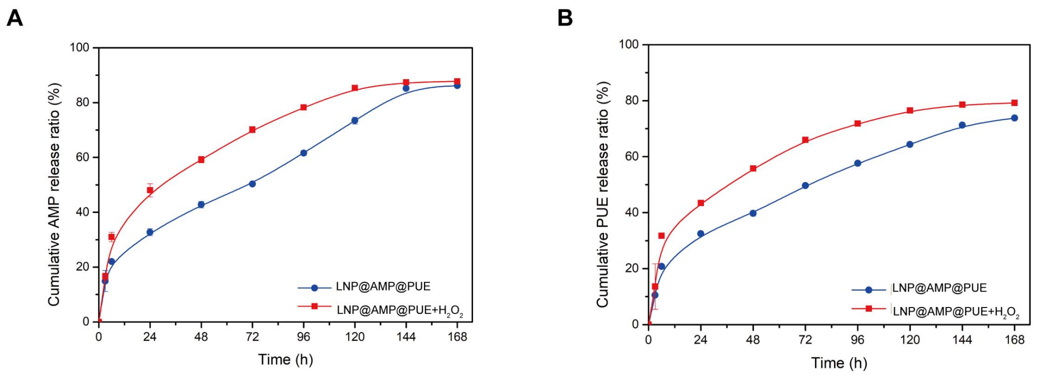

Figure S3. The cumulative release curves of AMP and PUE in the LNP@AMP@PUE. A) The release rate of AMP in LNP@AMP@PUE. B) The release rate of PUE in LNP@AMP@PUE.

Table S1: List of the main materials

Experimental reagent name	Specification	Company
silk fibroin (SF)	silkworm cocoon	/
glycidyl methacrylate (GM)	98.5%	Macklin
recombinant type III collagen	/	Ttrautec
methacrylic anhydride (MA)	AR	Macklin
4-(hydroxymethyl) phenylboronic acid pinacol ester (PBAP)	AR	Macklin
dichloromethane (CH_2Cl_2)	AR	Macklin
carbonyl diimidazole（CDI）	AR	Macklin
ethyl acetate	AR	Macklin
dextran (DEX)	Mw=10,000	Macklin
dimethyl sulfoxide（DMSO）	AR	Macklin
4-dimethylaminopyridine (DMAP)	AR	Macklin
antibacterial peptide (AMP)	/	Apeptide
puerarin（PUE）	95%	Macklin
hydrogenated soy lecithin（HSPC）	98%	AVT
cholestenone（CHO-HP）	98%	AVT
ethanol	AR	Aladdin

Table S2: Primer sequences for Real-time quantitative PCR

Target gene	Primers (5'–3'; F = forward; R = reverse)
Human-CD80	Forward: AAACTCGCATCTACTGGCAAA
	Reverse: GGTTCTTGTACTCGGGCCATA
Human-CD206	Forward: GGGTTGCTATCACTCTCTATGC
	Reverse: TTTCTTGTCTGTTGCCGTAGTT
Human-ARG-1	Forward: GTGGAAACTTGCATGGACAAC
	Reverse: AATCCTGGCACATCGGGAATC
Human-IL10	Forward: CCTCCGTCTGTGTGGTTTGAA
	Reverse: CACTGCGGTAAGGTCATAGGA
Human-iNOS	Forward: GTTCCAGATGAATACTGGCAGTC
	Reverse: GCAACTGAACACTATCTTTCCCT
Human-IL1β	Forward: ATGATGGCTTATTACAGTGGCAA
	Reverse: GTCGGAGATTCGTAGCTGGA
Human-GAPDH	Forward: TGTGGGCATCAATGGATTTGG
	Reverse: ACACCATGTATTCCGGGTCAAT
Human-Vcam1	Forward: GGATCGCTCAAATCGGGTGA
	Reverse: GGTGACTCGCAGCCCGTA
Human-CD31	Forward: GTGCTCTATGCAAGCCTCCA
	Reverse: TTCGAGGTGGTGCTGATGTC
Human-VWF	Forward: GCAGTGGAGAACAGTGGTG
	Reverse: GTGGCAGCGGGCAAAC

Table S3: Data of antibodies used in IF

Antibody	Company	Code	Specificity	IF
CD31+VEGF	proteintech/abcam	ab281583/ab1316	Rabbit/Mouse	1:200/1:500
TGFβ1	proteintech	21898-1-AP	Rabbit	1:200
MPO	servicebio	GB11224-100	Rabbit	1:200
TNF-α	proteintech	66142-1-Ig	Mouse	1:200
IFN-γ	proteintech	15365-1-AP	Rabbit	1:200
IL4	bioss	bs-0581R	Rabbit	1:200
Caspase 3	Servicebio	GB115600-100	Rabbit	1:700
CD68	abcam	ab955	Mouse	1:500
Ki67	servicebio	GB151142-100	Mouse	1:500

Table S4: List of PUE-related target genes

CTD	SWIS	TCMSP
NOS3	F10	ESR1
HMOX1	CA2	AR
NFE2L2	F2	PTGS2
CASP3	ITGAV ITGB3	CA2
BCL2	PRKCG	GSK3B
GCLC	PRKCD	CAMSAP2
TNF	PRKCA	RELA
RELA	PRKCB	STAT3
CAT	PRKCZ	AKT1
ESR2	PRKCE	VEGFA
FYN	PRKCH	BCL2
ALB	ITGAV ITGB6	FOS
BAX	PRKACA	BAX
MAPK8	F7	CASP9
MTOR	SQLE	MMP2
NFKB1	AKT1	MMP9
NOS2	SLC5A2	TNFAIP6
PARP1	NQO1	JUN
PGR	NQO2	NOS2
PPARA	EGLN1	CASP3
PTGS2	KISS1R	NFKBIA
RARA	ADRA2C	CASP8
RPS6KB1	F9	BAD
TGFB1	TP53	SOD1
ACTA2	IKBKB	PRKCA
AKT1	ITGA2B ITGB3	TIMP2
BIRC5	ITGB1 ITGA5	HIF1A
CASP8	BACE1	FAS
CCN2	MCL1	PPARG
COL1A1	BCL2	VCAM1
COX1	BAD	MAPK9
CYP2E1	TOP1	BIRC5
EIF4EBP1	PTGS2	NOS3
FASLG	VCP	PLAT
GDNF	TNNC1 TNNT2 TNNI3	CYP19A1
GPER1	CA6	GSTP1
GSK3B	PIK3CA	CHEK2
GSR	MMP9	SELPLG
ICAM1	PLAU	AGTR1
IGF1	MMP2	AKR1B1
IL6	PTGES	IFNB1
MAPK1	ALDH2	PDGFA

MAPK3
NQO1
PML
PPARG
PRKCD
SLC25A4
SLC25A5
ACACA
ACACB
ACADM
ACHE
ACLY
ACOX1
ALDH1A2
AQP1
AQP3
AR
BMP2B
CDH1
COL3A1
COX2
COX3
CPT1A
CREB1
CYGB
CYP7A1
DSG3
EGF
ESR1
FASN
FBN1
FBN2
FGF1
FOS
GPI
GPT
HAS1
HK1
HMGCR
HSPB1
IFNG
IL1A
IL1B
INSR
IL2
DUSP3
CALM1
PPARG
TNF
LTB4R
ALOX12
TBXAS1
MAOA
EGFR
MGAM
HTR2A
HTR2C
ADORA2A
HSD17B1
ESRRA
ESRRB
ABCG2
ADORA1
MIF
TYR
PFKFB3
HSD17B2
CBR1
PTGS1
SLC6A2
ESR1
SELP
PTPRS
ABCB1
ALOX15
ALOX5
SELL
MAOB
ESR2
CDK9 CCNT1
CCNA2 CDK2
PDE4D
OPRM1
OPRK1
CA4
HIF1A
PON1
PTPN1
ACE2
GPT
PGP
JAK3
CDKN1B
CDKN2AIP
IFNA1
ITGB5
LEPR
PLIN2
F2R

KL	ACHE
LDHA	CYP19A1
LDLR	XDH
LIF	NOX4
MAPK9	CDK5R1 CDK5
MC1R	SLC29A1
MMP2	AKR1B1
MYC	CA7
NFKBIA	CA12
NOS1	
PFKP	
PGAM1	
PLOD3	
PPARGC1A	
PTGER1	
SIRT1	
SLC2A1	
SOD3	
SQSTM1	
SRD5A2	
SREBF1	
TBX1	
TERT	
TIMP1	
TLR4	
TPT1	
TXN	
TYRP1	
UCP2	
UGT1A1	
UGT1A10	
UGT1A3	
UGT1A6	
UGT1A7	
UGT1A8	
UGT1A9	
VEGFA	
XIAP	
